# Historical studies on the use of Rhubarb in Japan

**DOI:** 10.3389/fphar.2025.1726521

**Published:** 2026-02-10

**Authors:** Misato Ota, Toshiaki Makino

**Affiliations:** Department of Pharmacognosy, Graduate School of Pharmaceutical Sciences, Nagoya City University, Nagoya, Aichi, Japan

**Keywords:** laxative effect, medicinal history, origin, *Rheum palmatum*, *Rheum rhabarbarum*, Rhubarb, *Rumex madaio* (*Rumex daiwoo*)

## Abstract

**Background:**

Rhubarb product is the crude drug derived from the root or rhizome of *Rheum* or other related plants, and has been used since ancient times in Asian and European countries. However, the original plant species for Rhubarb product had been confused throughout its long history and across different areas.

**Methods:**

The present study aimed to identify the original plant species for Rhubab products used in Japan through histological analysis of medicinal literature, successive Japanese Pharmacopoeias, and the textbooks of crude drugs published from the mid-Edo period (1603–1868) to the early Showa era (1926–1989).

**Results:**

During the Edo period, *Rumex madaio* (synonym, *Rumex daiwoo*), a plant that grows wild in Japan, was initially recognized as the origin of Rhubarb product. However, our present study indicates that, after *Rheum rhabarbarum* was imported from continental China in the 16th century, it was cultivated as the origin of true Rhubarb product. Since Rhubarb product derived from *Rheum rhabarbarum* has weak laxative effects, it is reasonable to infer that strong laxative effects were not anticipated in Japan at that time. Meanwhile, Rhubarb product derived from *Rheum palmatum* was known in Japan as Russian Rhubarb. *Rheum palmatum* had spread from continental China to Europe *via* Russia, and had been introduced to Japan as a laxative when Dutch medicine was introduced in the late-Edo period. Since the Meiji era (1868–1912) in Japan, the use of Rhubarb product during the Edo period had been re-evaluated, and Rhubarb product in Japan had been registered in the Japanese Pharmacopoeia. However, the publications in the Meiji era were reluctant to recognize that the Rhubarb product known as *Toh*-Rhubarb used by Japanese traditional Kampo physicians had derived from *Rheum rhabarbarum*. As a result, the two Rhubarb products were distributed separately, apart from those derived from *Rheum palmatum*. One was used by Kampo physicians and derived from *Rheum* plants grown in continental China, and referred to as *Toh*-Rhubarb. The other, derived from *Rheum rhabarbarum,* was referred to as *Wa*-Rhubarb.

**Conclusion:**

Consequently, the names and origins of Rhubarb products had been changed after the Meiji era from those used during the Edo period.

## 1 Introduction

The rhizomes of *Rheum palmatum* L., *Rheum tanguticum* Maxim. ex Balf., *Rheum officinale* Baillon, and *Rheum coreanum* Nakai, belonging to the section Palmata, the family Polygonaceae, or their interspecific hybrids, are registered as Rhubarb in the current 18th edition of the Japanese Pharmacopoeia (The Society of Japanese Pharmacopoeia, 2021). The first three species originate from continental China, while the last one comes from the Korean Peninsula. In this paper, capitalized “Rhubarb” refers not to the plant but the medicinal product (crude drug) derived from the rhizomes or roots of *Rheum* or other related species, and “rhubarb” is used as the common name of the plants related to Rhubarb. The Rhubarb in *Shosoin* treasure house of *Todaiji* temple, where the medicinal substances had been collected during the Tang Dynasty (618–907) and has been preserved until now, was the rhizome of *Rheum palmatum* or *Rheum tanguticum* (Shibata, 1991), suggesting that Rhubarb had been imported from continental China to Japan before the Tang Dynasty. On the other hand, *Rheum rhabarbarum* L. (synonym, *Rheum undulatum* L.), belonging to the section Rhapontica, was also imported from continental China and cultivated in the mid-Edo period (1603–1868) (Matsuoka, 1961; Kubo et al., 1997), and the rhizome of this species may have been used for clinical purposes. *Rheum rhabarbarum* is grown in the Korean Peninsula, and its rhizome had been used as Rhubarb (Ishidoya, 1925). Its rhizome was also used as a folk medicine in Qinghai and Gansu Provinces in continental China (Namba et al., 1989). While *Rheum coreanum* grows in the northern part of the Korean Peninsula, it is reported that this species has never been used for medicinal purposes in the Korean Peninsula (Ishidoya, 1925). It is unclear when *Rheum rhabarbarum* had been imported to Japan and whether it was expected to have the same medicinal properties as *Rheum palmatum* or *Rheum tanguticum*, etc.

Rhubarb has two main effects: purgative and improving blood circulation. The former is mainly due to the action of sennosides (Tsukui et al., 1974; Oshio et al., 1978), which are present in the rhizomes of four species prescribed in the Japanese Pharmacopoeia, but not in that of *Rheum rhabarbarum* (Choi et al., 2011). While there are several reports on the ameliorating effects of *Rheum rhabarbarum* on blood circulation. The aqueous extract of *Rheum rhabarbarum* rhizome induced relaxation of the phenylephrine-precontracted rat aorta in concentration-dependent manners (Moon et al., 2006). The active ingredients in *Rheum rhabarbarum* rhizome extract have been reported to be stilbene compounds such as piceatannol and may be mediated through an endothelium-dependent nitric oxide signaling pathway (Oh et al., 2007; Yoo et al., 2007). The production of cGMP was increased in a dose-dependent manner when the aqueous extract of *Rheum rhabarbarum* rhizome was added to human umbilical vein endothelial cells, suggesting that it suppresses the vascular inflammatory process *via* endothelium-dependent NO/cGMP signaling (Moon et al., 2006). In addition, *Rheum rhabarbarum* rhizome contains stilbene compounds such as desoxyrhapontigenin and rhapontigenin, which show significant inhibitory effects on blood aggregation induced by arachidonic acid and collagen, but piceatannol did not (Ko et al., 1999).

Several studies have also reported that the rhizomes of the plants belonging to the section Palmata have beneficial effects on blood circulation. The dried root of *Rheum officinale* significantly reduced the phenylhydrazine-induced reactive oxygen species (ROS) staining intensity in zebrafish and improved the thrombosis inhibition rate by upregulating the expression of endothelial NO synthase (eNOS, NOS3) mRNA (Zhang et al., 2022). The roots of *Rheum palmatum*, *Rheum officinale*, and *Rheum tanguticum* all showed *in vitro* thrombin inhibitory activity, and especially the roots of the first two species having higher activity (Liang et al., 2022).

In Europe, *Rheum rhabarbarum* and its hybrids with *Rheum rhaponticum* or *Rheum hybridum* (known as *Rheum × hybridum*, synonym *Rheum × rhabarbarum*) are cultivated (Tanhuanpää et al., 2019), with the petiole being used as an edible ingredient in jams and other dishes. However, it was not used for medicinal purposes. On the other hand, Rhubarb from continental China has been known in Europe as a purgative, but the history of its spread is not fully understood.

In the present study, we reviewed the ancient Japanese literature from the mid-Edo period to the early Showa era (1926–1989) to elucidate the reason for the change of original plant species whose rhizome was historically used as Rhubarb, from the perspectives of importation and the usage of *Rheum rhabarbarum*. In addition, the differences in the history of using Rhubarb for medicinal purposes in Europe and Japan were also clarified. Since there was no distinction between roots and rhizomes during the Edo period, the medicinal part of the original *Rheum* plants were referred to as roots in this article. After the distinction between root and rhizome was established during the Meiji era (1868–1912), this article follows the descriptions in the original literature.

## 2 Materials and methods

The descriptions about Rhubarb in medicinal literature, successive Japanese pharmacopoeias, and textbooks of crude drugs from the mid-Edo to the early Showa era were reviewed using the databases of National Diet Library Digital Collections https://dl.ndl.go.jp, Waseda University Library’s collection of Japanese and Chinese classics https://www.wul.waseda.ac.jp/kotenseki/index.html, and Kyoto University Rare Materials Digital Archive https://rmda.kulib.kyoto-u.ac.jp/en.

## 3 Results

### 3.1 Descriptions of the plants related to Rhubarb in the Edo period

The morphological descriptions of the plant used for Rhubarb in the medicinal literature of the Edo period are summarised in Table 1, and the characteristics of plants considered as the original plants of Rhubarb in Japan are summarized in Table 2. “Illustrated Materia Medica (*Zukai Honzo*)” (Shimotsu, 1680) mentioned that the original plant for Rhubarb was similar to *yotei*, the Japanese common plant name of *Rumex japonicus* Houtt. (*gishigishi* in the present) at that time, but the plant body was much larger, reaching 1.8–2.1 m in height and having a sour taste. “Explanation and Commentary on Records of Medicinal Properties (*Yakushoki Benkai*)” (Okamoto, 1699) described the leaves of the original plant for Rhubarb were round in shape, whereas “Illustrated Sino-Japanese Encyclopedia of the Three Realms (*Wakan Sansai Zue*)” (Terashima, 1824) described that they were rough, long, thick, and emerging from all four directions. “Brief Compendium of Medicinal Herbs (*Yakuso Rhakuhu*)” (Kyakuika, 1793) described the leaves of the original plant for Rhubarb were similar to those of *Arctium lappa* L., whereas “Illustrated Explanation of Poisonous Plants (*Yudoku Somoku Zusetsu*)” (Kiyohara, 1827), “Illustrated Manual of Materia Medica (*Honzo Zufu*)” (Iwasaki, 1830a), and “Investigation of Crude Drugs in Classical Prescriptions (*Koho Yakuhin Ko*)” (Naito, 1840) described that they were similar to those of *Paulownia tomentosa* (Thunb.) Steud. Since the leaves of *Arctium lappa* and *Paulownia tomentosa* are different in appearance, the original plants of Rhubarb were confused during this period.

**TABLE 1:** The characteristics of the original plants of Rhubarb described in Japanese literature during the Edo period (1603–1868).

Year	Literature title	Characteristics of the original plants of Rhubarb	Predicted plant
1680	Illustrated Materia Medica (*Zukai Honzo*: 図解本草) (Shimotsu, 1680)	The leaves and stems are similar to *yotei*, but it is about 1.8–2.1 m tall, fragile, and has a sour taste. The leaves are rough, long, and thick. The red roots are similar to those of *yotei*. The larger roots resemble teacups and measure about 60 cm in length	The plant used as the original plant for Rhubarb in China
1699	Explanation and Commentary on Records of Medicinal Properties (*Yakushoki Benkai:* 薬性記弁解) (Okamoto, 1699)	Rhubarb is a plant that has round leaves	*Rheum rhaponticum* or *Rheum rhabarbarum*
1763	Classification and Appraisal of Things (*Butsurui Hinshitsu*: 物類品隲) (Hiraga, 1763)	The Chinese species are the better, with leaves measuring about 60 cm and large roots with brocade patterns. The seed does not germinate when planted, but if the root is cut into dozens of pieces and planted, it will germinate	​
1793	Brief Compendium of Medicinal Herbs (*Yakuso Rhakuhu*: 薬草略譜) (Kyakuika, 1793)	The leaves are similar to burdock and are glossy. The stems are reddish, and the reddish-yellow flowers bloom in July and August. Its product is commonly called “brocade patterned-Rhubarb.” This species is found in Jiangshu Province and Ibukiyama mountain	*Rumex madaio*
1824	Illustrated Sino-Japanese Encyclopedia of the Three Realms *(Wakan Sansai Zue*: 和漢三才図会) (Terashima, 1824)	This is similar to *yotei*, 90 cm tall, with leaves emerging from all four directions, rough, long, and thick. Yellow flowers bloom in March, black fruits appear in May, and roots are harvested in August	*Rumex madaio*
1827	Illustrated Explanation of Poisonous Plants (*Yudoku Somoku Zusetsu*: 有毒草木図説) (Kiyohara, 1827)	Since it does not germinate from seed, it is better to plant the roots. The leaves are broad, similar to paulownia leaves, and have no serrations. The stems grow to be 1.5–1.8 m tall in the summer. They have alternate leaves and ear-shaped flowers, and then they bear fruit	*Rheum rhabarbarum*
1830	Illustrated Manual of Materia Medica *(Honzo Zufu:* 本草図譜) (Iwasaki, 1830a)	Rhubarb sprouts in the spring from planted roots, and the leaves are thick, slightly similar to paulownia. The round stems grow to 1.2–1.5 m in summer, and the stalks are purplish. It is smaller than *yotei* and has yellow-green flowers. Later, it produces three-lobed fruits. Its shape is similar to buckwheat fruits. The roots will be weak if the flower stems are not removed. Around February, the basal part of stem should be picked, cut into small pieces, and planted. The root shape is similar to that of pokeberry root, with a reddish-black skin and a yellowish-red interior that produces a yellow juice. *Rheum palmatum* The third species is *Rheum palmatum*. Palmatum means palm of the hand, and the leaves are similar to palms. The stem is about 2.4 m long and has spikes of flowers that are similar in color to meat. The leaves are about 60 cm, some have light green to purple patches on the stem, etc. Leaves have 3–5 lobes and may be rolled. The roots are tuberous and branched. There is also sweet mucilage. It is said that the roots have the same effect on diarrhea as Rhubarb that we usually use	*Rheum rhabarbarum*
1830	Illustrated Manual of Materia Medica (*Honzo Zufu*: 本草図譜) (Iwasaki, 1830b)	There are two kinds of illustrations of rhubarb in the Dutch literature. One type is produced in Europe and is a rhubarb introduced from continental China. The other species, from Russia, is similar in shape to the Chinese species but has serrated leaves similar to curly mustard leaves. This species is said to have a strong laxative effect	​
1840	Investigation of Crude Drugs in Classical Prescriptions (*Koho Yakuhin Ko*: 古方薬品考) (Naito, 1840)	Rhubarb sprouts in the spring from planted roots. Its glossy leaves are similar to paulownia leaves. In the summer, the stems grow to 90–120 cm, and light green, *yotei*-like flowers bloom. The roots are yellow and similar to pokeberry roots	*Rheum rhabarbarum*

**TABLE 2:** The characteristics of plants considered as the original plants of Rhubarb in Japan.

Species	*Rheum palmatum L.* (Editors of Flora of China, Chinese Academy of Sciences, 1998)	*Rheum officinale* Baillon (Editors of Flora of China, Chinese Academy of Sciences, 1998)	*Rheum rhabarbarum* L. (Makino, 1940; Editors of Flora of China, Chinese Academy of Sciences, 1998)	*Rumex madaio* Makino (Makino, 1940)	*Rumex japonicus* Meisn. (Makino, 1940)	*Rumex acetosa* L. (Makino, 1940)
Height	1.5–2 m	1.5–2 m	1.5 m	About 1 m	60–100 cm	50–80 cm
Root	​	The inside is yellow	It is yellow and thickened	It is yellow, large, and thickened	It is yellow, slightly large	It is branched and yellow
Stem	It is erect and hollow on the inside	It is hollow on the inside and covered in short white hairs	The inside is hollow	Erect, greenish purple with many longitudinal furrow lines, slightly branched at the top	Erect	Erect. Elongate, cylindrical, with longitudinal ridges. Green, usually tinged with reddish purple
Leaf	The leaves are palm-shaped and semi-five lobed. It has five veins, and the upper surface is coarsely hairy while the underside is covered with short hairs	The leaves are nearly circular and rarely broadly ovate. Their apexes are acuminate, and their bases are nearly heart-shaped. The leaves are palmately lobed with shallow lobes and 5–7 veins. The upper surface is shiny and hairless and underside has light brown, short hairs	Ovate, with 5–7 veins and wavy margins. Lower leaves are heart-shaped	The leaves at the base are long and large, and the stipules are alternate and ovate-lanceolate. The upper leaves gradually decrease in size and become bracts	Long, oblong oval with an acute head and a round or slightly wedge-shaped base	Oval, acute-headed, with a slightly arched base. Stem leaves alternate, lanceolate-elliptic, arched at the base
Petiole	It is cylindrical in shape, covered with hairs	It is roughly cylindrical in shape, with an edge line and short hairs covering its surface	Purple and long. It has shallow grooves on its upper surface and a rounded dorsum	Long petiole	Long petiole	Long petiole
Flower	It has large, conical, branched inflorescences. The flowers are small and purple-red, sometimes white	It has large, conical, branched inflorescences. The flowers are green to yellowish white	It is arranged in a compound raceme with a ring of small, yellowish-white flowers	Conical inflorescences are rare, and the flower axils are slightly curved. Flowers are green or purplish green and small. They grow in rings around the axis, but the rings are often far apart	It is arranged in a compound raceme with a ring of small, light green flowers. The inflorescence is interspersed with leafy bracts	The flowers branch off the stems and form conical spikes. It forms a ring of small, pale green or greenish-purple flowers

**FIGURE 1:**
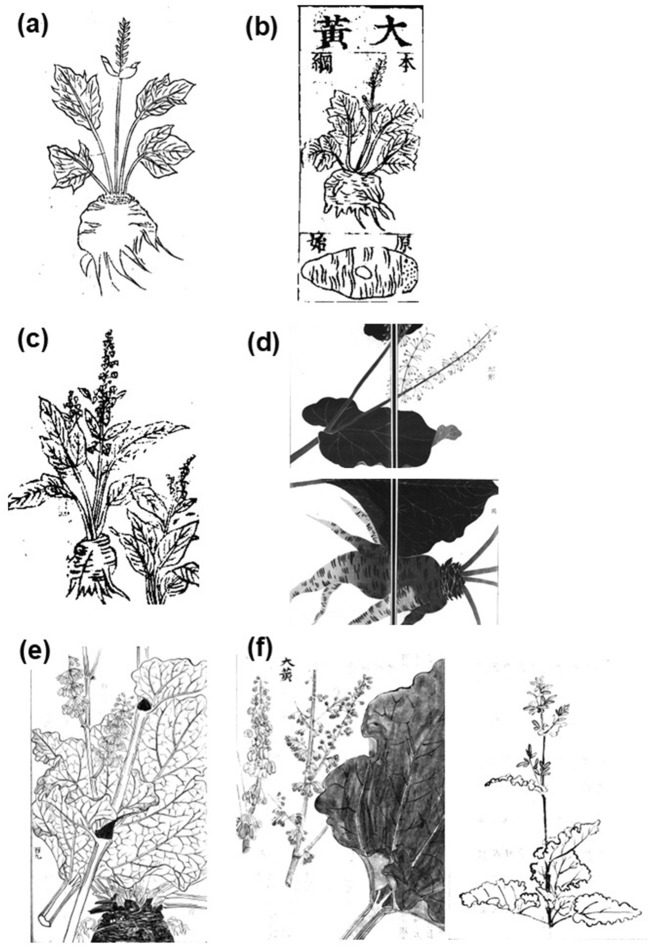
Attached illustrations of rhubarb in “Illustrated Materia Medica (*Zukai Honzo*: 図解本草)” (Shimotsu, 1680) **(a)**, “Complete Compendium of Materia Medica of Wide Benefit (*Koeki Honzo Taisei*: 広益本草大成)” (Okamoto, 1698) **(b)**, “Illustrated Sino-Japanese Encyclopedia of the Three Realms (*Wakan Sansai Zue*: 和漢三才図会)” (Terashima, 1824) **(c)**, “Illustrated Manual of Materia Medica (*Honzo Zufu*: 本草図譜)” (Iwasaki, 1830a) **(d)**, “Investigation of Crude Drugs in Classical Prescriptions (*Koho Yakuhin Ko*: 古方薬品考)” (Naito, 1840) **(e)**, and “Recorded Hearings on the Compendium of Materia Medica (*Honzo Komoku Kibun*: 本草綱目紀聞)” written by Mizutani (n.d.) **(f)**.

The color of flowers of the original plant for Rhubarb was described as yellow in “Brief Compendium of Medicinal Herbs (*Yakuso Rhakuhu*)” (Kyakuika, 1793), yellow in “Illustrated Sino-Japanese Encyclopedia of the Three Realms (*Wakan Sansai Zue*)” (Terashima, 1824), yellow-green in “Illustrated Manual of Materia Medica (*Honzo Zufu*)” (Iwasaki, 1830a), and light green in “Investigation of Crude Drugs in Classical Prescriptions (*Koho Yakuhin Ko*)” (Naito, 1840). Since only the first description has a reddish tint, it is possible that the plant species described in “Brief Compendium of Medicinal Herbs (*Yakuso Rhakuhu*)” was different from the other three literature. “Brief Compendium of Medicinal Herbs (*Yakuso Rhakuhu*)” (Kyakuika, 1793) described that the plant had reddish stems. “Illustrated Manual of Materia Medica (*Honzo Zufu*)” (Iwasaki, 1830a) described that its petiole was purple. “Illustrated Manual of Materia Medica (*Honzo Zufu*)” (Iwasaki, 1830a) and “Investigation of Crude Drugs in Classical Prescriptions (*Koho Yakuhin Ko*)” (Naito, 1840) described that its fruit resembled that of *Fagopyrum esculentum* Moench, and its root resembled that of *Phytolacca americana* L. The illustrations in the medicinal literature depicted three types of leaves: palm-shaped (Figures 1a,b), long-leafed (Figure 1c), and wavy-edged (Figures 1d–f).

**FIGURE 2:**
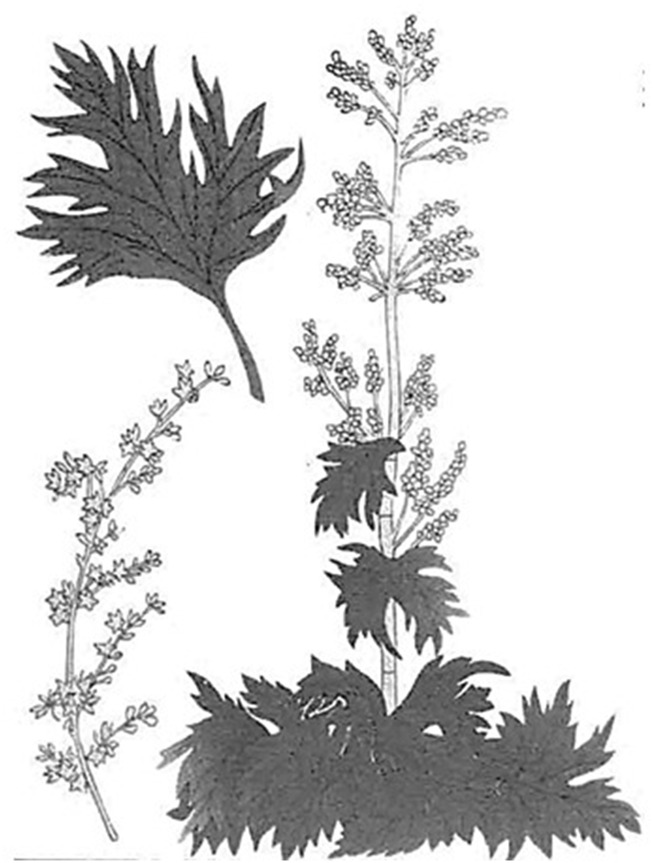
Attached illustration of *Rheum palmatum* in “Illustrated Manual of Materia Medica (*Honzo Zufu*: 本草図譜)” (Iwasaki, 1830a).

The first description of *Rheum palmatum* appeared in “Illustrated Manual of Materia Medica (*Honzo Zufu*)” (Iwasaki, 1830a). This literature depicted the original plant for Rhubarb having palmate leaves and red flowers in the attached figure (Figure 2), which was consistent with *Rheum palmatum* (Table 2). Another edition of this literature (Iwasaki, 1830b) stated that two types of plants related to Rhubarb were listed in Dutch literature: one from Europe that was introduced from China, and one from Russia that was similar in shape to the Chinese type but had serrated leaves like those of *Brassica juncea* subsp. *integrifolia* (West) Thell. It stated that the Russian type differed from the original plant for Rhubarb considered in Japan because it had serrations of the leaves and resembled *Rheum palmatum*, as shown in the attached figure (Figure 2).

### 3.2 Plants related to Rhubarb grown in Japan during the Edo period

Plants related to Rhubarb in Japan (Japanese rhubarb) could be classified into six types: cultivated rhubarb, *yotei*-rhubarb, *do*-rhubarb, *toh*-rhubarb, *wa*-rhubarb, and *shin*-rhubarb (Supplementary Table S1). *Yotei* (羊蹄) was presumably *Rumex japonicus*. *Do* (土) means soil. *Toh* (唐) is a Japanese adjective that means derived from continental China by respecting the Tang dynasty. *Wa* (和) means Japan, and *shin* (真) means true.

Both “Correction of Misunderstandings about Materia Medica (*Honzo Bengi*)” (Endo, 1681) and *Honzo Wage* (Masatsugu, 1712) stated that the original plant for Rhubarb grown in Japan did not coincide with the description of “Compendium of Materia Medica (*Honzo Komoku* in Japanese, 1596)” (Li, 2004). However, *Toeki Hengyoku Honzo* (Takada, 1683) stated that they were the same.

“Correction of Misunderstandings about Materia Medica (*Honzo Bengi*)” (Endo, 1681) stated that Japanese Rhubarb had the best fragrance, similar to Chinese Rhubarb. “Complete Book of Drug Processing (*Hosha Zensho*)” (Ino, 1702) described that the areas where Japanese rhubarb was cultivated were Yamato (part of present-day Nara prefecture), Yamashiro, and Tango (part of present-day Kyoto prefecture). “Secret Formulas of Materia Medica (*Honzo Hiketsu*)” (Author anonymous, 1826) stated that Japanese Rhubarb was the variety of Chinese Rhubarb called “brocade patterned Rhubarb”. “Investigation of Crude Drugs in Classical Prescriptions (*Koho Yakuhin Ko*)” (Naito, 1840) stated that this variety had been imported from continental China during the Kyoho era (1716–1736). “Explanations of Crude Drugs in Classical Prescriptions (*Koho Yakusetsu*)” (Ujita, 1795) stated that the plants from continental China were easy to grow in Japan. However, “Secret Formulas of Materia Medica (*Honzo Hiketsu*)” (Author anonymous, 1826) stated that the plants imported from continental China were a different species because they could not be grown in Japanese soil. Therefore, it is considered that the rhubarb that could be grown in Japan had been imported from continental China during the Kyoho era, but that this plant must be different from the original Chinese rhubarb. “Complete Book of Agriculture (*Nogyo Zensho*)” (Miyazaki, 1697) described that *toh*-rhubarb was cultivated at Yamashiro and other places, and that this type was the representative of Japanese rhubarb. It stated that the leaves of *toh*-rhubarb were round and thick, the stems were slightly red, similar to those of *Farfugium japonicum* (L.) Kitam., and the roots were similar to those of *wa*-rhubarb, but that the species of *toh*-rhubarb was different from *wa*-rhubarb.

“Recorded Hearings on Materia Medica (*Honzo Kibun*)”*,* written by Ono (n.d.), stated that *Toh-*Rhubarb was imported from continental China during the Kyoho era, which was similar to the description of Japanese Rhubarb. Meanwhile, “Explanations of Crude Drugs in Classical Prescriptions (*Koho Yakusetsu*)” (Ujita, 1795) stated that *Do*-Rhubarb had been mistakenly called *Toh*-Rhubarb. “Brief Compendium of Medicinal Herbs (*Yakuso Rhakuhu*)” (Kyakuika, 1793) described that the products derived from *toh*-rhubarb were similar to those from *sambo*.

**FIGURE 3:**
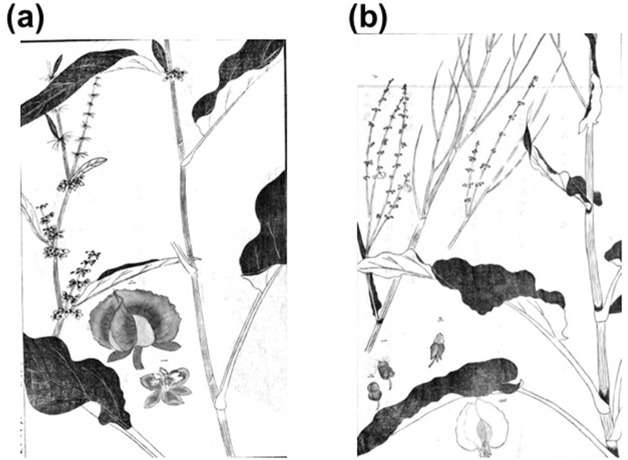
Attached illustrations of *gishigishi* **(a)** and *suiba* **(b)** in “Illustrated Explanation of Plants (*Somoku Zusetsu*: 草木図説)” (Iinuma, 1856).

“Correction of Misunderstandings about Materia Medica (*Honzo Bengi*)” (Endo, 1681) stated that *yotei*-rhubarb was grown at the riversides and in rice fields, and its Japanese common name is *shinoko*, *gishigishi*, or *no*-rhubarb. “Explanation and Commentary on Records of Medicinal Properties (*Yakushoki Benkai*)” (Okamoto, 1699) stated that *yotei*-rhubarb has long and slender leaves, had been mistakenly called as Rhubarb, and was also known as *gishigishi*. “Recorded Hearings on the Compendium of Materia Medica (*Honzo Komoku Kibun*)” written by Mizutani (n.d.), described that *yotei-*rhubarb was sometimes called *ohoshi* (large *gishigishi*), and resembles *sambo*, but was larger than *sambo*. “Illustrated Explanation of Plants (*Somoku Zusetsu*)” (Iinuma, 1856) depicted that the leaves of *gishigishi* were long, and its numerous small flowers were arranged in whorls, and its inflorescence had leaf-like bracts, which were consistent with the characteristics of *Rumex japonicus* (Figure 3a). Therefore, *Yotei*-Rhubarb refers to the root of *gishigishi* (*Rumex japonicus*), and its root was used as a substitute for Rhubarb.

“Complete Book of Drug Processing (*Hosha Zensho*)” (Ino, 1702), *Yakuro Honzo* (Katsuki, 1727), and “Pocket Compendium of Essential Materia Medica (*Shuchin Honzo Shun*)” (Hirazumi, 1790) described that the majority of Rhubarb sold in drugstores was derived from the root of *yotei*-rhubarb. “Study of Crude Drugs for Daily Use (*Nichiyo Yakuhinko*)” (Shibata, 1810) stated that *yotei-*rhubarb had once been considered the original plant for Rhubarb, and that the Rhubarb appearing in the past prescriptions had been derived from the root of *yotei*-rhubarb. “Investigation of Crude Drugs in Classical Prescriptions (*Koho Yakuhin Ko*)” (Naito, 1840) stated that *Yotei-*Rhubarb should only be used externally.

**FIGURE 4:**
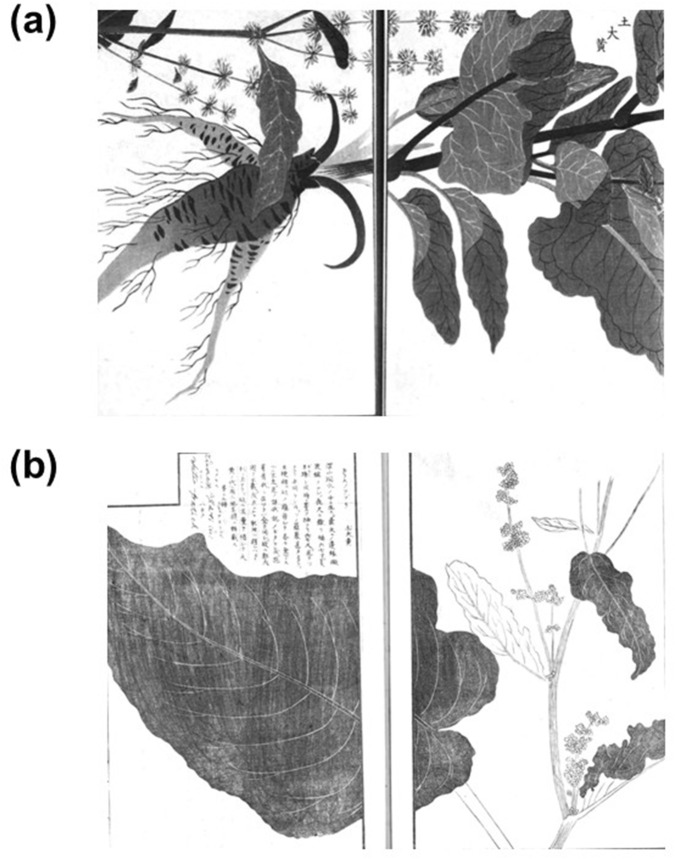
Attached illustrations of *do*-rhubarb in “Illustrated Manual of Materia Medica (*Honzo Zufu*: 本草図譜)” (Iwasaki, 1830a) **(a)** and “Illustrated Explanation of Plants (*Somoku Zusetsu*: 草木図説)” (Iinuma, 1856) **(b)**.

“Elucidation of Compendium of Materia Medica (*Honzo Komoku Keimo*)” (Ono, 1805), “Augmented Edition of the Primer on the Palm (*Zoho Shuhan Hatsumo*)” (Fujii, 1823), “Illustrated Explanation of Plants (*Somoku Zusetsu*)” (Iinuma, 1856), and “Illustrated Manual of Materia Medica (*Honzo Zufu*)” (Iwasaki, 1830a) mentioned that *do*-rhubarb was the another name of *karasunoabura.* “Ippondo’s Selection of Crude Drugs (*Ippondo Yakusen*)” (Kagawa, 1738) stated that *do*-rhubarb has shorter, rounder, and larger leaves than *yotei-*rhubarb, similar to the leaves of the original plant for Rhubarb, but that its edges were not chipped and its color is not the same. “Secret Formulas of Materia Medica (*Honzo Hiketsu*)” (Author anonymous, 1826) stated that *do*-rhubarb was called *ushinosita,* and its leaves resembled those of *Nicotiana tabacum* L. (tobacco). The attached illustration of *do-*rhubarb in “Illustrated Manual of Materia Medica (*Honzo Zufu*)” (Iwasaki, 1830a) and “Illustrated Explanation of Plants (*Somoku Zusetsu*)” (Iinuma, 1856) depicted those characteristics (Figure 4).

“Correction of Misunderstandings about Materia Medica (*Honzo Bengi*)” (Endo, 1681) and “Complete Compendium of Materia Medica of Wide Benefit (*Koeki Honzo Taisei*)” (Okamoto, 1698) mentioned that *do-*rhubarb resembled Japanese rhubarb species, but the color was more reddish, and that the Japanese common name of *do-*rhubarb was *sambo.* “Secret Formulas of Materia Medica (*Honzo Hiketsu*)” (Author anonymous, 1826) stated that *sambo* resembles *Ricinus communis* L. However, “Elucidation of Compendium of Materia Medica (*Honzo Komoku Keimo*)” (Ono, 1805) states that Li Shizhen, the author of “Compendium of Materia Medica (*Honzo Komoku* in Japanese, 1596)” (Li, 2004), had been incorrect in stating *sambo* was *do*-rhubarb, and that *sambo* had been known as *suiba* in Japanese (*Rumex acetosa* L.) and can be found in the aquatic plants section. The illustrated characteristics of *suiba* depicted in “Illustrated Explanation of Plants (*Somoku Zusetsu*)” (Iinuma, 1856) matched those of *Rumex acetosa,* having narrow, elongated leaves and branched, conical inflorescences (Figure 3b), suggesting that *sambo* and *suiba* are different plant species.

“Explanations of Crude Drugs in Classical Prescriptions (*Koho Yakusetsu*)” (Ujita, 1795) stated that *do*-rhubarb was sold in drugstores as *Wa*-Rhubarb, while “Compilation and Commentary on the Compendium of Materia Medica (*Honzo Komoku Sanso*)” (So, 1798) mentioned that wild Japanese rhubarb was not *shin*-rhubarb but *do*-rhubarb, revealing the confusion over the species names. Furthermore, “Newly Revised and Augmented Dutch Mirror of Medicine (*Shintei Zoho Oranda Yakkyo*)” (Udagawa, 1830) noted that unscrupulous merchants sold *Do*-Rhubarb mixed with *yotei* root, indicating that *Do*-Rhubarb was sometimes mixed with *Yotei*-Rhubarb.

“Ippondo’s Selection of Crude Drugs (*Ippondo Yakusen*)” (Kagawa, 1738) stated *Do*-Rhubarb was poor quality and should not be used. “Essential Knowledge for Using Medicines (*Yoyaku Suchi*)” (Matsuoka, 1726) noted that *Do*-Rhubarb had the same efficacies as that of *Yotei*-Rhubarb. “Illustrated Manual of Materia Medica (*Honzo Zufu*)” (Iwasaki, 1830a) stated that *Do*-Rhubarb also had a purgative effect. “Inquisitive Materia Medica (*Shitsumon Honzo*)” (Go, 1837) listed cooling and purging poison from whole body as the efficacies of *Do*-Rhubarb. “Illustrated Explanation of Plants (*Somoku Zusetsu*)” (Iinuma, 1856) noted that when using *Do*-Rhubarb in place of common Rhubarb, the dosage should be double. Therefore, the efficacies of *Do*-Rhubarb were similar but weaker than those of common Rhubarb.

“Minimumal 6 × 8 Materia Medica (*Hengyoku Rokuhati Honzo*)” (Kato, 1780) lists the original plants of *wa*-rhubarb as *gishigishi*, while “Illustrated Sino-Japanese Encyclopedia of the Three Realms (*Wakan Sansai Zue*)” (Terashima, 1824) lists it as *yotei* or *suiba* with large roots. “Study of Crude Drugs for Daily Use (*Nichiyo Yakuhinko*)” (Shibata, 1810) stated that most items sold as *Wa*-Rhubarb are actually *Do*-Rhubarb, the mixture of shaved *Shin*-Rhubarb and *Do*-Rhubarb. It also stated that *Yotei*-Rhubarb (*No*-Rhubarb) might be regarded as the low-quality product of *Wa*-Rhubarb. However, either *Do*-Rhubarb or *Yotei*-Rhubarb should not be used as common Rhubarb. Meanwhile, “Study of Japanese Rhubarb (*Nihon Daioko*)” (Shimizu, 1859) stated that *wa*-rhubarb is called *ohoshi* and is different from Chinese rhubarb. Therefore, *Wa*-Rhubarb referred to either products derived from *gishigishi* or *suiba*, *Do*-Rhubarb, or the mixture of shaved *Shin*-Rhubarb and *Do*-Rhubarb.

“Clarifying Confusions about Crude Drugs (*Yakuhin Benwaku*)” (Oguchi, 1754) stated that *shin*-rhubarb was derived from the Tang dynasty and was grown in Yamashiro, Yamato, and Tango areas. This description is similar to that of *toh*-rhubarb as mentioned above. While, “Investigation of Crude Drugs in Classical Prescriptions (*Koho Yakuhin Ko*)” (Naito, 1840) stated that *shin*-rhubarb refers to *do*-rhubarb. “Explanations of Crude Drugs in Classical Prescriptions (*Koho Yakusetsu*)” (Ujita, 1795) described that the leaves of *shin*-rhubarb resembled those of *do*-rhubarb and the roots of *shin*-rhubarb had a purple brocade pattern. “Elucidation of Compendium of Materia Medica (*Honzo Komoku Keimo*)” (Ono, 1805) noted that *Shin*-Rhubarb was not as good as the imported Rhubarb because they had not adapted to the Japanese soil, and that *shin*-rhubarb grown for more than 2 years had the same quality as Chinese rhubarb. “Compilation and Commentary on the Compendium of Materia Medica (*Honzo Komoku Sanso*)” (So, 1798) stated that due to the mild efficacies, *Shin*-Rhubarb should not be used in emergency situations. “Clarifying Confusions about Crude Drugs (*Yakuhin Benwaku*)” (Oguchi, 1754) stated that although imported Rhubarb had better quality, it was acceptable to use *Shin*-Rhubarb if imported Rhubarb from continental China was in short supply. These statements indicate that although *Shin*-Rhubarb was considered to have weaker efficiencies than imported Rhubarb, it was acceptable when imports were scarce.

**FIGURE 5:**
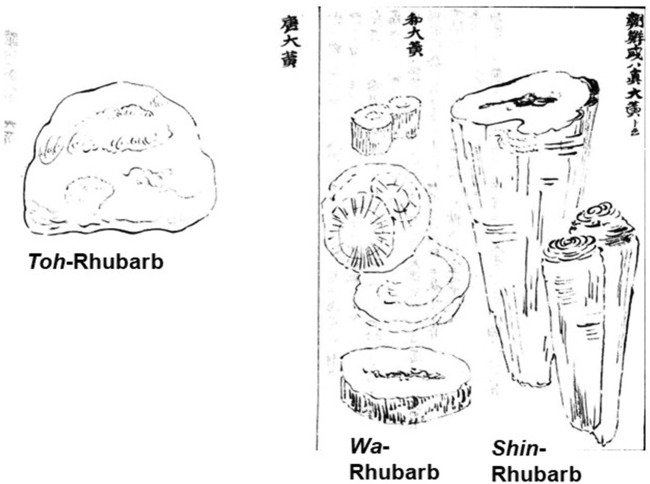
Attached illustrations of *Toh-, Wa-,* and *Shin-*Rhubarb in “Recorded Hearings on the Compendium of Materia Medica (*Honzo Komoku Kibun*: 本草綱目紀聞)” written by Mizutani (n.d.).

Furthermore, the literature in this era generally stated that *Shin*-Rhubarb possessed the brocade pattern characteristic of common Rhubarb. However, the attached illustration in “Recorded Hearings on the Compendium of Materia Medica (*Honzo Komoku Kibun*)” written by Mizutani (n.d.) showed that *Shin*-rhubarb had a swirl-like pattern, while *Wa*-Rhubarb and *Toh*-Rhubarb did not display this pattern (Figure 5).

Both “Study of Crude Drugs for Daily Use (*Nichiyo Yakuhinko*)” (Shibata, 1810) and “Illustrated Manual of Materia Medica (*Honzo Zufu*)” (Iwasaki, 1830a) described that the other names for *Shin*-Rhubarb and *Do*-Rhubarb were Korean Rhubarb. “Study of Japanese Rhubarb (*Nihon Daioko*)” (Shimizu, 1859) stated that Rhubarb produced in Japan was the same as that imported from the Korean Peninsula in the past, hence it was called Korean rhubarb.

### 3.3 Rhubarb imported from continental China during the Edo period

**FIGURE 6:**
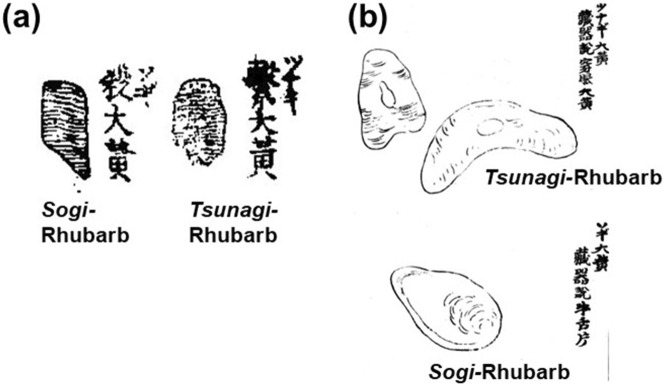
Attached illustrations of *Sogi-* and *Tsunagi-*Rhubarb in “Illustrated Sino-Japanese Encyclopedia of the Three Realms (*Wakan Sansai Zue*: 和漢三才図会)” (Terashima, 1824) **(a)** and “Recorded Hearings on the Compendium of Materia Medica (*Honzo Komoku Kibun*: 本草綱目紀聞)” written by Mizutani (n.d.) **(b)**.

Two types of Rhubarb were imported from continental China: *Sogi-* and *Tsunagi-* (*Sengan-*) Rhubarb (Supplementary Table S2). In “Illustrated Materia Medica (*Zukai Honzo*)” (Shimotsu, 1680), *Sogi*-Rhubarb was superior to *Tsunagi*-Rhubarb. “Correction of Misunderstandings about Materia Medica (*Honzo Bengi*)” (Endo, 1681) stated that *Sogi*-Rhubarb was the product made by cutting Rhubarb at an angle and then drying it, and that *Tsunagi*-Rhubarb was made by threading Rhubarb through a rope and drying it like a string of pearls, mentioning the difference in preparation methods. Both types of Rhubarb were painted in the illustrations of “Illustrated Sino-Japanese Encyclopedia of the Three Realms (*Wakan Sansai Zue*)” (Terashima, 1824) and “Recorded Hearings on the Compendium of Materia Medica (*Honzo Komoku Kibun*)” written by Mizutani (n.d.) (Figure 6). The perception that *Sogi*-Rhubarb was superior had remained largely unchanged until the early 18th century. However, “Augmented Edition of the Lingbao Medicinal Properties, Efficiency, and Toxicity (*Zoho Reiho Yakusho Nodoku*)” (Manase, 1698) regarded *Tsunagi* as superior. “Essential Knowledge for Using Medicines (*Yoyaku Suchi*)” (Matsuoka, 1726) stated that *Sogi*-Rhubarb was not true Rhubarb and was derived from *yotei*-rhubarb (*gishigishi*), and that *Tsunagi*-Rhubarb (*Sengan*-Rhubarb described in the ancient medicinal literature) was true Rhubarb. Namely, high-quality Rhubarb product was *Sogi* in the 17th century. By the 18th century, it had changed to *tsunagi*. *Sogi*-Rhubarb in the 18th century was derived from *yotei-*rhubarb (*gishigishi*).

“Ippondo’s Selection of Crude Drugs (*Ippondo Yakusen*)” (Kagawa, 1738) described that since the cow’s tongue-like type of Rhubarb was produced in Japan while the brocade-patterned type was made in China, the cow’s tongue-like type was considered as made in Japan among the *Sogi*-Rhubarb. Furthermore, “Minimumal 6 × 8 Materia Medica (*Hengyoku Rokuhati Honzo*)” (Kato, 1780) stated that *Sogi*-Rhubarb derived from *gishigishi* must not be used.

In the 19th century, “Elucidation of Compendium of Materia Medica (*Honzo Komoku Keimo*)” (Ono, 1805) stated that *Sogi*-Rhubarb had once been imported, but no longer was. “Augmented Edition of the Primer on the Palm (*Zoho Shuhan Hatsumo*)” (Fujii, 1823), citing Dutch theory, stated that *Sogi*-Rhubab had originated from Chinese mountains, while *sengan* (*tsunagi*) had come from Russia, incorporating a European perspective.

### 3.4 Views on Rhubarb during the Meiji era (1868–1912)

The descriptions of Rhubarb during the Meiji era are summarized in Supplementary Table S3. “Recorded Hearings of Daily Lectures on Materia Medica” (Ermerins, 1878) stated that Russian Rhubarb was native to continental China and was considered the products with high-quality. The first textbook of pharmacognosy (Wigand, 1888) in Japan noted that Chinese Rhubarb was derived from *Rheum officinale*, distinct from what was called *toh*-rhubarb. Meanwhile, European rhubarb included both *Rheum rhabarbarum* and *Rheum palmatum*. “Practical Methods for Medicinal Identification” (Sano, 1889) stated that European Rhubarb was inferior in quality compared to Chinese Rhubarb. The Supplement to the Japanese Pharmacopoeia revised edition (Kashimura and Ise, 1891) noted that while the original plant for Chinese Rhubarb was once considered *Rheum palmatum*, *Rheum officinale* was the primary original species at that time. It also noted that *Toh*-Rhubarb was used by traditional Chinese medicine practitioners, but it was derived from a different type of *Rheum* plant that is typically rotten and softened. This book also described that although the characteristic actinocyte and medullary patterns of Rhubarb are unknown, common Rhubarb in Japan was considered distinct from Chinese Rhubarb. In the textbook of Pharmacognosy (Shimoyama, 1904), the original plant for Chinese Rhubarb was defined as two species, *Rheum officinale* and *Rheum palmatum*, while European Rhubarb no longer included descriptions of *Rheum palmatum*. This textbook also stated that Japanese Rhubarb produced in Tsugaru area was the best, which resembled Austrian Rhubarb and was likely derived from *Rheum rhabarbarum*, but that the original plants of *Toh*-Rhubarb had remained unknown—a description that had not been updated from previous editions.

As stated in the “Preface to the Complete Works of Yukichi Fukuzawa” (Fukuzawa, 1897), the importation of Chinese Rhubarb had ceased approximately seven or eight years ago, and the price of Rhubarb suddenly soared. Consequently, the utilization of *Wa*-Rhubarb resulted to treat the onset of gastrointestinal disorders. “Concise Pharmacognosy” (Shimazaki, 1909) described that the plant commonly referred to *w*a-rhubarb was *Rumex japonicum* or *Rumex aquaticus*, etc. Meanwhile, “Pocket Pharmacognosy” (Nishimura, 1911) suggested that *wa*-rhubarb might be *Rheum rhabarbarum*.

In 1896, Tomitaro Makino stipulated that *do*-rhubarb was *Rumex madaio* Makino, the species he had previously designated, and that it was synonymous with *madaio* (Makino designated *shin*-rhubarb as *madaio*) (Makino, 1896). Concurrently, the “General Flora of Great Japan” (Saita, 1897) identified *do*-rhubarb as the same *madaio*, but attributed its original species to *Rumex aquaticus* L. var. *japonicus* Meisn.

### 3.5 Views on Rhubarb during the Taisho era (1912–1926)

The descriptions of Rhubarb during the Taisho era are summarized in Supplementary Table S4. The “Complete Compendium of Modern Pharmacy” (Ito, 1913) stated that Japanese Rhubarb was derived from *Rheum rhabarbarum* and that produced in Tsugaru area had high quality. The “Cultivation and Marketing of Important Medicinal Plants” (Bandai, 1918) noted that rhubarb cultivated in Tsugaru area was referred to as *toh*-rhubarb. The “Annotated Complete Collection for Pharmacist Examination Questions and Answers” (Onda, 1918) suggested that the original species of *Toh*-Rhubarb might be *Rheum rhabarbarum*.

The fourth edition of the Japanese Pharmacopoeia (Nagao, 1924) listed the item name *Toh*-Rhubarb for the first time, but its origin was listed as the Chinese *Rheum* plant without specifying the species. Meanwhile, that books outside the Japanese Pharmacopoeia, such as “Cultivation of Medicinal Plants for New Drugs” (Matsuda, 1920) and “Concise Lectures for Drug Merchants” (Maruyama, 1923) stated that the original plant for *Toh*-Rhubarb was *karadaio*, namely *Rheum rhabarbarum*. Note that the Han-letter for *karadaio* is identical to that for *Toh*-Rhubarb; *karadaio* refers to the plant, while *Toh*-Rhubarb refers to the medicinal product (crude drug), making them homonyms for different substances. Regarding the effect of *Toh*-Rhubarb, “Materia Medica for Drug Merchants” (1923) (Koyama, 1923) stated *Toh*-Rhubarb was used as a laxative and stomachic. Practical Guide or the Fourth Edition of the Japanese Pharmacopoeia for Physicians with Prescriptions (Nagao, 1924) stated that *Toh*-Rhubarb had the same medicinal effects as common Rhubarb and was mainly used as a raw material for medicines. Furthermore, “Concise Lectures for Drug Merchants” (Hino, 1925) noted that *Toh*-Rhubarb contained the component emodin.

“Annotated Complete Collection for Pharmacist Examination Questions and Answers” (Onda, 1918) stated that *Wa*-Rhubarb was derived from *Rumex daiwoo* Makino. Meanwhile, “Cultivation of Medicinal Plants for New Drugs” (Matsuda, 1920) stated that *Wa*-Rhubarb was derived from *Rheum rhabarbarum*.

### 3.6 Views on Rhubarb during the Showa era (1926–1989)

The descriptions of Rhubarb during the Showa era are summarized in Supplementary Table S5. The Register of Domestic Products (Kokusanshinkokai, 1928) stated that *Toh*-Rhubarb and *Wa*-Rhubarb were the same products, but their original plants differed slightly. While many texts mention *Rheum rhabarbarum* as the original plant for *Toh*-Rhubarb, the Pharmacognosy textbook (Shimoyama, 1943) and the “Latest Classified Compilation of Pharmaceutical Products” (Yoshimatsu, 1947) described that *Rheum rhabarbarum* might not be the original plant for *Toh*-Rhubarb. Conversely, these texts distinguished *Wa*-Rhubarb as originating from *Rheum rhabarbarum*. During this period, the origin of *Rheum rhabarbarum* was recorded as Siberia. Subsequently, the Sixth Edition of the Japanese Pharmacopoeia (Japan Pharmaceutical Association, 1951) listed items derived from *Rheum rhabarbarum* as *Wa*-Rhubarb. The Seventh Edition (Pharmaceutical and Medical Device Regulatory Science Society of Japan, 1961) maintained this entry, but the Eighth Edition (Pharmaceutical and Medical Device Regulatory Science Society of Japan, 1971) removed *Wa*-Rhubarb from the list.

Regarding the medicinal effects of *Toh*-Rhubarb and *Wa*-Rhubarb, the “Supplementary Notes on Kampo Medicines” (Nakayama, 1929) stated that Westerners used Turkish Rhubarb as a laxative, but when Japanese used it, it caused severe abdominal pain, so Japanese must use *Toh*-Rhubarb. This suggests *Toh*-Rhubarb may have been better suited to the Japanese constitution. Meanwhile, “Makino’s Illustrated Flora of Japan” (Makino, 1940) stated that *Toh*-Rhubarb, meaning Chinese Rhubarb, was mistaken for true Rhubarb, i.e., *Shin*-Rhubarb; however, *Toh*-Rhubarb was not true Rhubarb and had no medicinal value. “Latest Classified Compilation of Pharmaceutical Products” (Yoshimatsu, 1947) indicated that *Wa*-Rhubarb was used as a substitute for Rhubarb or *Toh*-Rhubarb as a laxative or stomachic, but its laxative effect was considered the mildest among its counterparts, and that rhubarb grown in Nara Prefecture had a strong laxative effect; however, it had also been reported to cause abdominal pain. “Illustrated Guide to Medicinal Plants” (Murakoshi, 1952) stated that *Wa*-Rhubarb can be used as a substitute for true Rhubarb. A small amount of *Wa*-Rhubarb had been used to treat indigestion, while a large amount had been used as a laxative in powder or decoction form. The recommended daily dosage ranges from 2 to 4 g.

In “Medicinal Plants for Home Knowledge” (Kyushu Medicinal Plant Research Society, 1929), *Do*-Rhubarb was referred to as *Shin*-Rhubarb, and its original species was called *karasunoabura*, with its medicinal properties equivalent to common rhubarb. “Makino’s Illustrated Flora of Japan” (Makino, 1940) assigned the species *Rumex daiwoo* as *do*-rhubarb, stating that the species name ‘Daiwoo’ was based on the fact that it was once mistaken for medicinal rhubarb, which Japanese name meant ‘true rhubarb’ but it was originally misidentified and was not actually a true rhubarb. This book also described that while suggesting that the *ohoshi* mentioned in the *Engishiki* (the book describing about laws and customs in Japan published in 927) could be *shin*-rhubarb, the author refuted his own theory, stating it was unlikely to have been cultivated about 1,000 years ago.

“Studies on Japanese Medicines” (Shimizu, 1941) stated that *Wa*-Rhubarb was the root of *Rheum rhabarbarum*, and that *Rumex daiwoo* was used for a fake *Toh*-Rhubarb. In “Latest Classified Compilation of Pharmaceutical Products” (Yoshimatsu, 1947) stated that the roots of *Rumex japonicus* and *Rumex acetosa* were also treated as the origin of Rhubarb in Japan, but were said to cause severe abdominal pain.

### 3.7 Trends in the use of Rhubarb around the world and in Japan from the 16th to the 19th century

Table 3 summarizes global trends in Rhubarb from the 16th to the 19th century. In 1510, Portugal colonized Goa, India, and began using Rhubarb, one of the Indian medicines. Rhubarb was considered an effective remedy for constipation and diarrhea due to its purgative and astringent properties. Many European conscripts suffered from cholera and other gastrointestinal diseases; therefore, Rhubarb was very useful to the Portuguese (Walker, 2011). At the same period, Rhubarb was carried on Portuguese ships traveling from continental China to Japan as part of the Nanban trade (Oka, 2008). On the other hand, historical records indicate that Rhubarb was exported from continental China to Russia in 1568 and 1654, with the latter export being larger in volume (Romaniello, 2016). Records indicated that Rhubarb was exported from continental China to Japan in 1698 (Huang, 2004). However, by the late 17th century, Japan began promoting cultivation of the original plant of Rhubarb as part of efforts to domestically produce crude drugs (Xu, 2018). Although Rhubarb was imported from continental China during the 18th and 19th centuries (Miyashita, 2018), it was extremely expensive, forcing reliance on domestic substitutes. While there were records of Korean Rhubarb being imported in 1642 (Tashiro, 1999), imports had become rare after the 18th century (Iwanaga, 1800).

**TABLE 3:** Trends in the use of Rhubarb around the world and in Japan from the 16th to the 19th century.

Century	Trends in the use of Rhubarb around the world	Trends in the use of Rhubarb in Japan
The 16th	In 1510, Portugal colonized Goa, India, and began using Indian spices and medicines. Rhubarb was prized for its healing properties throughout India, but was costly to collect and distribute due to its uncertain origin. Rhubarb was considered an effective remedy for constipation and diarrhea due to its purgative and astringent properties. Many European conscripts suffered from cholera and other gastrointestinal diseases, so rhubarb was very useful to the Portuguese. As Portuguese merchants expanded into India, they supplied Europe with Indian rhubarb, which was brought by ships that circumnavigated Africa. Rhubarb from India was shipped to Russia, where it was referred to as “Chinese” or “Russian” Rhubarb upon its arrival in Europe (Walker, 2011)	Rhubarb was carried on Portuguese ships traveling from China to Japan as part of the Nanban trade. The price of Rhubarb doubled once it reached Japan. Trade records state that there is an infinite supply of Rhubarb in China (Oka, 2008)
	In 1568, there was a record that rhubarb had been exported from China to Russia (Romaniello, 2016)	​
The mid-17th	In 1649, Nicholas Culpeper of England noted that boiling Rhubarb reduces its purgative effect. He also recommended soaking it in white wine overnight and drying it over a low heat (Chang, 2005)	In 1642, various Korean crude drugs were imported to Japan, and there is a record of Rhubarb from Korea being presented to Iemitsu Tokugawa (Tashiro, 1999)
	In 1654, Rhubarb was exported in large quantities from China to Russia, and it was then exported to Europe (Romaniello, 2016)	In 1698, a record exists of rhubarb being transported on a ship from Ningbo in China to Japan (Huang, 2004)
The last-17th	​	There was a strong shift towards the domestic production of crude drugs, with six cultivated varieties (Cnidium rhizome, Alisma rhizome, Angelica root, Angelica dahurica root, Rhubarb and Sparganium rhizome) being traded on the Edo and Osaka markets (Xu, 2018)
The 18–19th	​	From 1735 to 1862, Rhubarb was included on the list of drugs that were imported from China (Miyashita, 2018)
	​	In May 1857, imported Chinese Rhubarb cost 420 sen (100 sen = 1 Japanese yen) per 600 g, making it expensive. Japanese rhubarb was therefore often used instead. Imports of Rhubarb from Korea were also rare (Iwanaga, 1800)

Regarding the use of Rhubarb, Nicholas Culpeper of England noted in 1649 that boiling Rhubarb reduces its purgative effect. He also recommended soaking it in white wine overnight and drying it over a low heat. Thus, the knowledge that boiling weakens its laxative properties had reached England (Chang, 2005), though they employed different processing methods than those used in China.

## 4 Discussion

### 4.1 The original plant species for Rhubarb during the Edo period

Descriptions in medicinal literature from the Edo period indicated that there were two main types of rhubarb leaves: 1. round and broad, resembling paulownia leaves, and lacking serrations; 2. long and thick, resembling burdock leaves, and resembling *yotei* (*Rumex japonicus*). The attached illustration shows three leaf types: A. palmate leaves (Figures 1a,b); B. long leaves (Figure 1c); and C. wavy-edged leaves (Figures 1d–f). We selected plants native to China and Japan from Flora of China (Author anonymous, 1998) and the first edition of Makino’s Illustrated Flora (Makino, 1940) (Table 2) that were possibly used as the original plant for Rhubarb in Japan. We then compared these plants with descriptions in ancient medicinal literature. The results suggest that A is likely *Rheum officinale*, B is likely *Rumex madaio*, and C is likely *Rheum rhabarbarum*. Furthermore, based on the descriptions, classification 1 is most likely *Rheum rhabarbarum*, and classification 2 is most likely *Rumex madaio*. The predicted plant names are listed in Table 1.

The attached illustrations in “Illustrated Materia Medica (*Zukai Honzo*)” (Shimotsu, 1680) and “Complete Compendium of Materia Medica of Wide Benefit (*Koeki Honzo Taisei*)” (Okamoto, 1698) may be quoted from Chinese medicinal literature of “Compendium of Materia Medica (*Honzo Komoku* in Japanese, 1596)” (Li, 2004), and they coincide with the leaf shape of *Rheum officinale*, a plant used in China. On the other hand, the former text was a quotation from “Newly Revised Materia Medica (*Shinshu Honzo* in Japanese, 659)” (Su, 2013) and “Illustrated Classic of Materia Medica (*Zukyo Honzo* in Japanese, n.d.)” described in the “Materia Medica Arranged According to Pattern (*Shorui Honzo* in Japanese, 1108)” (Tang, 1971). The description of the leaves resembles *yotei*, and this literature also contained the description “sour.” It is unclear to which part of the plant this refers, but the petioles of the edible rhubarb (*Rheum × hybridum*) and the leaves of *Rumex acetosa*, are said to taste sour because they contain high levels of oxalic acid (Cojocaru et al., 2019; Korpelainen and Pietiläinen, 2020). Meanwhile, there are no reports of this for *Rheum officinale*. Although this is the plant considered to be the original plant species of Rhubarb in China, when compared with subsequent materia medica descriptions, it is similar to *Rumex madaio*. However, as there are no reports on the taste of *Rumex madaio*, it cannot be identified based on taste alone.

In “Illustrated Sino-Japanese Encyclopedia of the Three Realms (*Wakan Sansai Zue*)” (Terashima, 1824), the leaf shape depicted in the illustration and accompanying text resembles that of *Rumex madaio*. However, the yellow flower color is closer to that of *Rheum rhabarbarum*. In “Brief Compendium of Medicinal Herbs (*Yakuso Rhakuhu*)” (Kyakuika, 1793), written at the end of the 18th century, the flowers are described as reddish, which is a characteristic closer to that of *Rumex madaio*. Since plants in the *Rheum* genus are self-incompatible and easily produce hybrids, it is possible that *Rumex madaio* hybrids also began to exist around the end of the 18th century.

The plant’s morphology is described in detail in “Illustrated Manual of Materia Medica (*Honzo Zufu*)” (Iwasaki, 1830a), including the purple petioles and yellowish flowers, which coincide with those of *Rheum rhabarbarum*. The attached illustration clearly shows the flowers arranged in a whorl, confirming its identity as *Rheum rhabarbarum*. Furthermore, “Investigation of Crude Drugs in Classical Prescriptions (*Koho Yakuhin Ko*)” (Naito, 1840) depicts it more accurately, showing the wavy leaves and whorled flowers characteristic of *Rheum rhabarbarum*.

Meanwhile, “Illustrated Manual of Materia Medica (*Honzo Zufu*)” (Iwasaki, 1830a; Iwasaki, 1830b) introduces the third species, *Rheum palmatum*, noting that it has similar anti-diarrheal effects to the rhubarb commonly used today. This suggests that *Rheum palmatum*, also known as medicinal rhubarb, was likely used as a substitute at that time. Additionally, the description in the Dutch text cited by “Illustrated Manual of Materia Medica (*Honzo Zufu*)” (Iwasaki, 1830b), which stated that the Russian variety resembled the Chinese one in shape but had serrated edges like crepe mustard, is believed to refer to *Rheum palmatum*. This indicates that, at the time, it was believed that *Rheum palmatum* was Russian, not Chinese, in origin.

Thus, it can be inferred that, until the late 18th century, two species—*Rumex madaio* and *Rheum rhabarbarum*—were recognized as rhubarb in Japan. Thereafter, only *Rheum rhabarbarum* was designated as rhubarb. Conversely, since *Rheum palmatum* was identified as Russian rhubarb, it is unlikely that it was ever considered true Chinese rhubarb.

### 4.2 The original plant species for Rhubarb grown in Japan during the Edo period

Li Shizhen wrote *do*-rhubarb was *sambo* in “Compendium of Materia Medica (*Honzo Komoku* in Japanese, 1596)” (Li, 2004). The Japanese mistakenly interpreted this as referring to *suiba* (*Rumex acetosa*). The character “酸” (*sam* in Japanese, which means sour taste) in *sambo* (酸模) suggests sourness. It is possible that they perceived it as *suiba*, whose leaves are sour. Consequently, some texts identified *do*-rhubarb as *suiba*. However, the attached illustrations of *do*-rhubarb in “Illustrated Manual of Materia Medica (*Honzo Zufu*)” (Iwasaki, 1830a) and “Illustrated Explanation of Plants (*Somoku Zusetsu*)” (Iinuma, 1856) depicted leaves that were round and broad with small, spaced-apart flowers arranged in whorls. This morphology coincides with that of *Rumex madaio*, which was later named by Tomitaro Makino (Makino, 1896). Assuming *do*-rhubarb refers to *Rumex madaio*, there is no contradiction with the description in “Secret Formulas of Materia Medica (*Honzo Hiketsu*)” (Author anonymous, 1826) leaves resembling those of *Nicotiana tabacum*. *Do*-rhubarb likely referred to *Rumex madaio*, but the original plant remained unknown due to the absence of Japanese name at the time and its sale under an incorrect name in drugstores. However, this investigation could not determine what is *sambo*, said to resemble *Ricinus communis*.

### 4.3 The original plant species for *Toh*-, *Wa*-, and *Shin*-Rhubarb during the Edo period

The original plant species for *Toh*-, *Wa*-, and *Shin*-Rhubarb are summarized in Table 4. Some literature stated that the original plant of Rhubarb cultivated in Japan was imported from continental China. “Recorded Hearings on Materia Medica (*Honzo Kibun*)”, written by Ono (n.d.), provides a similar description of *toh*-rhubarb. This suggests that, since the Chinese pronunciation at the time was “*Tang*” (read as “*Toh*” in Japanese), the rhubarb introduced from continental China was named “*toh*-rhubarb.” Since *Rheum rhabarbarum* is native to continental China, the rhubarb introduced from China was probably this species. The description of *toh*-rhubarb in “Complete Book of Agriculture (*Nogyo Zensho*)” (Miyazaki, 1697) mentioned leaves that were round and thick, resembling *Farfugium japonicum*, and stems that were slightly red. These characteristics coincide with those of *Rheum rhabarbarum* and present no contradiction. However, according to Kinoshita’s opinion (Kinoshita, 2015), the first rhubarb introduced to Japan was *Rheum rhaponticum*. Since the stems of *Rheum rhaponticum* are not red like those of *Rheum rhabarbarum* (Makino, 1940; Libert and Englund, 1989; Author anonymous, 1998), we concluded that the rhubarb introduced to Japan was derived from the latter species.

**TABLE 4:** The summary of original plant species used as *To*-, *Wa*-, *Shin*- and *Do*-Rhubarb in Japan.

Period	*Toh*-Rhubarb	*Wa*-Rhubarb	*Shin*-Rhubarb	*Do*-Rhubarb
Edo (1603–1868)	*Rheum rhabarbarum*	*Rumex japonicus* *Rumex acetosa* *Rumex madaio*	*Rumex madaio* (Before the widespread cultivation of *Rheum rhabarbarum*) *Rheum rhabarbarum*	*Rumex madaio*
Meiji (1868–1912)	Unknown plants	*Rheum rhabarbarum* *Rumex* plants	Not in use	*Rumex madaio*
Taisho (1912–1926)	*Rheum* plants grown in continental China (The fourth edition of the Japanese Pharmacopoeia) *Rheum rhabarbarum* (Outside of the Japanese Pharmacopoeia)	*Rheum rhabarbarum* *Rumex madaio*	Not in use	Not in use
Showa (1926–1989)	*Rheum* plants grown in continental China (The fifth edition of the Japanese Pharmacopoeia)	*Rheum rhabarbarum* (The sixth and seventh editions of the Japanese Pharmacopoeia)	Not in use	Not in use

The term ‘*wa*-rhubarb’ can refer to any of the following three species, all of which are native to Japan: *gishigishi* (*Rumex japonicus*), *suiba* (*Rumex acetosa*), and *do*-rhubarb (*Rumex madaio*). This suggests that, in Japan, the term ‘*wa*-rhubarb’ referred to all rhubarb produced domestically, excluding imports from China.

In most Japanese medicinal literature, ‘*shin*-rhubarb’ and '*toh*-rhubarb’ (*Rheum rhabarbarum*) refer to the same plant. This suggests that the rhubarb recognised in Japan was known as ‘*shin*’ ('true’ in Japanese) rhubarb. However, before *Rheum rhabarbarum* was widely cultivated in Japan, other large-leaved rhubarbs, namely *Rumex madaio*, were recognised as Japanese rhubarb, which likely caused some confusion.

Medicinal literature on the Edo period described *Shin*-Rhubarb as having a brocade pattern. The front illustration in “Recorded Hearings on the Compendium of Materia Medica (*Honzo Komoku Kibun*)” written by Mizutani (n.d.) and the only text to include an attached diagram, shows a pattern that resembles swirling lines. It is possible that this pattern was referred to as ‘brocade’. However, the pattern is unclear and absent from the larger illustration in the background. Furthermore, the *Toh*-Rhubarb illustration also lacks this pattern. This suggests that the type of brocade pattern associated with Rhubarb was not well understood in Japan at that time.

### 4.4 The medicinal effects of *Toh*-, *Wa*-, *Shin*-, *Do*-, and *Yotei*-Rhubarb during the Edo period

Japanese Rhubarb was highly prized for its aroma and potent medicinal properties, especially for healthy people during the 17–18th centuries. In contrast, *Yotei*-Rhubarb was considered an inferior substitute. *Do*-Rhubarb had a mild laxative effect, and *Shin*-Rhubarb was also less potent than Chinese Rhubarb. Therefore, the Japanese Rhubarb that was considered to have strong medicinal effects in Japan at that time is not thought to be *Yotei-*, *Do-,* or *Shin-* Rhubarbs. However, since the original plant of *Shin*-Rhubarb was confused with either *Rheum rhabarbarum* or *Rumex madaio*, it is possible that only the former was considered high quality. On the other hand, *Rumex* plants have long been valued in folk medicine in Asia, America, and Europe. They have been used as hemostatic, diuretic, and laxative agents. Furthermore, since *Rumex* plants have been reported to contain compounds similar to those found in common Rhubarb, such as anthraquinones, flavonoids, tannins, and stilbenes (Li et al., 2022), treating them as Rhubarb was not incorrect despite their potency.

“Ippondo’s Selection of Crude Drugs (*Ippondo Yakusen*)” (Kagawa, 1738) stated that although Chinese Rhubarb had been intensely dried using fire, it still had a stronger purgative effect than Japanese Rhubarb, and that Chinese Rhubarb was better for its purgative action than Japanese Rhubarb. This literature also described that Japanese Rhubarb was better for reducing blood stasis and joint toxicity without causing severe diarrhea, even when taken long-term. It had a milder effect, making it suitable for weak people, as it cures the patient without necessarily causing purgation. In other words, Rhubarb was probably more highly valued in Japan for its ability to reduce blood stasis than for its strong laxative effect. However, this is the only Japanese medicinal literature that mentions reducing blood-stasis properties of Japanese Rhubarb; others only discuss its laxative properties and compare different types of Rhubarb. Since Japan mainly used products derived from *Rheum rhabarbarum*, it can be inferred that a strong laxative effect was not required. Additionally, since the *Rheum rhabarbarum* used in Japan had a naturally weak laxative effect, further heating was likely unnecessary. This is thought to be the reason why Rhubarb processing had not become widespread in Japan.

As mentioned in “Supplementary Notes on Kampo Medicines*”* (Nakayama, 1929), written in the Showa era, the type of Rhubarb that does not cause abdominal pain may have been well-suited to the Japanese constitution. Furthermore, while the reducing blood-stasis properties of Rhubarb derived from *Rheum rhabarbarum* are highly valued in Korea, Rhubarb with these properties appears to have been used in Japan since ancient times.

“Ippondo’s Selection of Crude Drugs (*Ippondo Yakusen*)” (Kagawa, 1738) stated that Japanese Rhubarb was suitable for weak individuals. However, other literature suggested that its potent effects made it unsuitable for healthy people, resulting in conflicting descriptions. The latter may simply have intended to assert the superiority of Japanese Rhubarb over Chinese varieties, but the reason for this remains unclear.

### 4.5 The reason *Rheum rhabarbarum* was introduced to Japan

The earliest recorded cultivation of a plant believed to be *Rheum rhabarbarum* in Japan was referenced in “Complete Book of Agriculture (*Nogyo Zensho*)” (Miyazaki, 1697). Although the literature published in the 19th century stated that this plant was introduced during the Kyoho era (1716–1736) (Naito, 1840), it had actually been present in Japan prior to that time. In continental China, the rhizome of *Rheum palmatum* and *Rheum officinale* were the major species used, but the rhizome of *Rheum rhabarbarum* was also used as folk medicine in the Provinces of Qinghai and Gansu (Namba et al., 1989). Furthermore, the rhizome of *Rheum rhabarbarum* was also used in the Korean Peninsula. Therefore, two possibilities can be considered for the introduction of *Rheum rhabarbarum* to Japan.

1. *Rheum* *rhabarbarum* may have been imported from the Korean Peninsula.
2. *Rheum* *rhabarbarum* may have been mistakenly imported from continental China as the original plant of true Rhubarb.

Regarding point (1), the records show that Rhubarb was imported from the Korean Peninsula in 1642 (Tashiro, 1999), which was just before the publication of “Complete Book of Agriculture (*Nogyo Zensho*)” (Miyazaki, 1697). Later, Rhubarb produced in Japan and *shin*-Rhubarb were sometimes referred to as 'Korean Rhubarb’, which also coincided with the imported variety from the Korean Peninsula. However, as Rhubarb was presented to the general, it is unlikely that its use spread widely throughout Japan.

Regarding (2), Chinese Rhubarb was imported into Japan in the 17th century as *Sogi-* and *Tsunagi-*Rhubarb. A variety of rhubarb cultivated in Japan was already referred to as *toh*-rhubarb at this time, meaning that products derived from *Rheum rhabarbarum* must have been introduced even earlier. Additionally, there are records of Portuguese ships bringing Rhubarb from continental China to Japan *via* the Nanban trade (Oka, 2008). These records noted that Rhubarb was plentiful in continental China and that exporting it to Japan doubled its price, suggesting a profit motive. Therefore, if financial gain was the goal, it would not be surprising if the wrong type of Rhubarb had been introduced.

At the same time, Portugal had colonised India and realised that true Rhubarb, derived from *Rheum palmatum*, was an extremely useful medicinal product for soldiers suffering from diseases such as cholera (Walker, 2011). Therefore, it seems unlikely that they would have sold it purely for profit. This suggests that *Rheum rhabarbarum* was most likely introduced from continental China *via* the Nanban trade. Furthermore, although the plant seeds would be required for the cultivation, as mentioned in “Classification and Appraisal of Things (*Butsurui Hinshitsu*)” (Hiraga, 1763), this plant germinates poorly from seed and is usually propagated by root division. Therefore, if some of the imported rhubarb roots were not completely dried, it might have been possible to produce seedlings.

Regarding *Sogi-* and *Tsunagi-* (*Sengan-*) Rhubarb, which had been imported from continental China since the 17th century, *Sogi* was considered the higher quality variety in the 17th century. However, by the late 18th century, *Tsunagi* had replaced it as the preferred variety, and *Sogi* was no longer available on the market. The reason for this shift was that high-quality *Sogi* ceased to be imported from continental China. Consequently, *yotei* (*gishigishi*) began to be processed into the same shape and sold as *Sogi* in Japan. Once this practice was recognised as counterfeit, *Tsunagi* came to be regarded as the superior product, resulting in a change to the standard of quality. Therefore, it can be inferred that Chinese *Sogi* disappeared from the market around the beginning of the 18th century, rather than at the end.

From the 16th century onward, rhubarb derived from the Chinese species *Rheum palmatum* spread throughout Europe *via* Russia and other routes. However, Japan began shifting towards producing Rhubarb domestically around the late 17th century. This occurred just before Chinese-produced *Sogi* disappeared from the market. The Rhubarb derived from *Rheum rhabarbarum,* which had been introduced to Japan earlier, was cultivated and regarded as Japanese Rhubarb. In contrast, the use of Rhubarb derived from *Rheum palmatum* had been very limited. Consequently, even after the introduction of Dutch medicine, Rhubarb derived from *Rheum palmatum* was referred to as “Russian Rhubarb,” suggesting that it was not considered the true Chinese Rhubarb.

On the other hand, since Rhubarb derived from *Rheum palmatum* was introduced to Japan from Europe during the adoption of Dutch medicine, it is thought that its use replaced Western medical laxatives. The Rhubarb preserved in the *Shosoin* repository was believed to be either *Rheum palmatum* or *Rheum tanguticum*, suggesting that true Rhubarb was imported to Japan at that time. However, the Rhubarb mainly used in Japan during the Edo period was derived from *Rheum rhabarbarum*. Therefore, it is possible that Rhubarb derived from *Rheum palmatum* traveled from continental China to Europe *via* Russia and then returned to Japan when Dutch medicine was introduced.

### 4.6 The original plant species of Rhubarb during the Meiji era

During the Meiji era, detailed descriptions of the characteristics of Rhubarb from various regions were provided. In the late 19th century, Russian Rhubarb was considered a high-quality variety originating from China; however, the original plant was not specified, suggesting that its origin was unknown. In the first textbook on Pharmacognosy translated by Gendo Oi in 1888 (Wigand, 1888), the origin of Chinese Rhubarb was identified as *Rheum officinale*, while *Rheum palmatum* and *Rheum rhabarbarum* were classified as European. By 1904, the pharmacognosy textbook (Shimoyama, 1904) stated that Chinese Rhubarb originated from two species: *Rheum officinale* and *Rheum palmatum*. This suggests that, during the early Meiji era, *Rheum palmatum* had not yet been recognized as the origin of Chinese Rhubarb. Since *Rheum palmatum* was transmitted from continental China to Russia and Europe, it is highly probable that Russian Rhubarb also referred to the product derived from *Rheum palmatum*.

*Rheum rhabarbarum* is native to Europe and was considered to be of inferior quality compared to the Chinese variety. *Toh*-rhubarb had been first described as *Rheum rhabarbarum* in the botanical literature published in 1897 (Saita, 1897), but subsequent references stated that the original plant was unknown. The pharmacognosy textbook (Shimoyama, 1904) suggested that rhubarb cultivated in Japan might be *Rheum rhabarbarum*. This implies that recognizing that European species *Rheum rhabarbarum* could not be the same as *toh*-rhubarb, led to the original species being designated as unknown. At the same time, it was suggested that the cultivated Japanese species, *wa*-rhubarb, might be *Rheum rhabarbarum*. This resulted in the two products derived from *toh-* and *wa-*rhubarb being treated as distinct.

Furthermore, the Supplement to the Japanese Pharmacopoeia Revised Edition (Kashimura and Ise, 1891) stated that *Toh*-Rubarb is rotten and softened, lacking brocade patterns. As mentioned above, this suggests that the nature of the brocade patterns themselves may not have been understood during the Edo period. It can also be inferred that *Toh*-Rhubarb was perceived as inferior in the Meiji era.

The “Preface to the Complete Works of Yukichi Fukuzawa” (Fukuzawa, 1897) mentioned gastrointestinal disorders caused by *Wa*-Rhubarb. This is likely because it was made from plants outside the *Rheum* genus, such as *Rumex japonicus* and *Rumex aquaticus*. For the aforementioned reasons, however, Rhubarb made in Japan derived from *Rheum rhabarbarum* had come to be called *Wa*-Rhubarb, which differs from *Wa*-Rhubarb in the Edo period (Table 4).

As the plant *do*-rhubarb did not have another common Japanese name, Tomitaro Makino gave it the Japanese common name *madaio* in 1896, along with the Latin name *Rumex madaio* (Makino, 1896). *Madaio* (真大黄) is an alternative reading of *shin*-rhubarb in Japanese. Specifically, he seemed to have considered *do*-rhubarb, a Japanese plant used as the origin of Rhubarb, to be *shin*-rhubarb. However, this study clarifies that, after the cultivation of *toh*-rhubarb became widespread, *shin*-rhubarb referred to *toh*-rhubarb. Therefore, it can be concluded that his designation was based on the prior theory, before the cultivation of *toh*-rhubarb became widespread.

### 4.7 The original plant species of Rhubarb during the Taisho era

During the Taisho era, many publications began attributing to *toh*-rhubarb to *Rheum rhabarbarum*. However, the fourth edition of the Japanese Pharmacopoeia (1920) (Choyokai Co, 1920) specified the original plant as a Chinese species of the genus *Rheum*. The official Pharmacopoeia appears to have avoided specifying plants with ambiguous origins. Some literature applied *Rheum rhabarbarum* to *wa*-rhubarb, while others applied *Rumex madaio* (Table 4). This suggests that *wa*-rhubarb, meaning Japanese rhubarb, was applied to the plant other than *Rumex japonicus* (*gishigishi*) and *Rumex acetosa* (*suiba*), both of which are unsuitable for use as the origin of Rhubarb.

### 4.8 The original plant species of Rhubarb during the Showa era

During the Showa era, there was a trend to return to the Meiji era’s definition of *Rheum rhabarbarum* as Japanese Rhubarb (*Wa*-Rhubarb) rather than *Toh*-Rhubarb, which was used by Kampo physicians during the Edo period. The Sixth and Seventh Editions of the Japanese Pharmacopoeia (Japan Pharmaceutical Association, 1951; Pharmaceutical and Medical Device Regulatory Science Society of Japan, 1961) no longer listed *Toh*-Rhubarb; instead, they designated *Rheum rhabarbarum*-derived products as *Wa*-Rhubarb (Table 4). However, subsequent editions omitted listing altogether. This may have been an attempt to acknowledge that *Rheum rhabarbarum* was cultivated in Japan, while denying the implication that the medicinal efficacy of Rhubarb could not be expected if the *Toh*-Rhubarb used in Japanese Kampo medicine was derived from *Rheum rhabarbarum*. In Tomitaro Makino’s Illustrated Flora of Japan (Makino, 1940), he asserted that *Toh*-Rubarb, derived from *Rheum rhabarbarum*, was not true Rhubarb and had no medicinal value. However, while Korean physicians used Rhubarb derived from *Rheum rhabarbarum*, Japanese physicians may not have recognized this practice. Nevertheless, *Rheum rhabarbarum* appears to have little value in modern Japanese medicine and was likely excluded from the Japanese Pharmacopoeia. This indicates that the focus of rhubarb in Japan has shifted towards its laxative properties.

Tomitaro Makino also revised the Latin name that he had previously given to *Rumex madaio*, changing it to *Rumex daiwoo*. This emphasised that the species had previously been mistakenly identified as the origin of Rhubarb, which is also written as *daiwoo* in Japanese. He also stated that *Rumex madaio* might have been cultivated about 1,000 years ago. However, our present study revealed it was probably not a cultivated variety, but rather a native Japanese species*,* similar to *Rumex japonicus* (*gishigishi*) and *Rumex acetosa* (*suiba*).

## 5 Conclusion

During the Edo period in Japan, *Rumex madaio*, a plant that grows wild in Japan, was initially recognized as the original plant for Rhubarb. However, our present study indicates that, after *Rheum rhabarbarum* was imported from China in the 16th century, it was cultivated as the true origin of Rhubarb. Since Rhubarb derived from *Rheum rhabarbarum* has weak laxative effects, it is reasonable to infer that strong laxative effects were not anticipated in Japan at that time. Meanwhile, Rhubarb derived from *Rheum palmatum* was known in Japan as Russian Rhubarb. *Rheum palmatum* spread from continental China to Europe *via* Russia and was believed to have been introduced to Japan as a laxative when Dutch medicine had been introduced.

Since the Meiji era, the use of Rhubarb during the Edo period had been re-evaluated, and Rhubarb produced in Japan had been included in the Japanese Pharmacopoeia. However, the literature in Meiji era were reluctant to acknowledge that the Rhubarb used by Kampo physicians was derived from *Rheum rhabarbarum*. As a result, the three types of Rhubarb had been distributed separately. The first was common Rhubarb derived from *Rheum palmatum* used in modern medicine; the second was used by Kampo physicians referring to *Toh*-Rhubarb; the third was *Wa*-Rhubarb produced in Japan derived from *Rheum rhabarbarum*. It is thought that, consequently, the names for Rhubarb differed after the Meiji era from those used during the Edo period.

## Data Availability

The original contributions presented in the study are included in the article/Supplementary Material, further inquiries can be directed to the corresponding author.
