# Supplementary material for: Historical studies on the use of Rhubarb in Japan
Source: Front Pharmacol. 2026 Feb 10;16:1726521. doi: 10.3389/fphar.2025.1726521 (PMC12929373; doi:10.3389/fphar.2025.1726521)
Supplement: Supplementary file 1 [file Table1.docx]

**SUPPLEMENTARY TABLE 1** Descriptions of rhubarb grown in Japan during the Edo period (1603–1868).

| **Year** | **Literature title** | **Descriptions** |
| --- | --- | --- |
| 1681 | Correction of Misunderstandings about Materia Medica (*Honzo Bengi*: 本草弁疑) (Endo, 1681) | **Rhubarb produced in Japan**: The rhubarb grown in Japan does not coincide with the description of Compendium of Materia Medica (Li, 2004) in terms of the color of its stems, leaves, and roots. The roots have the best fragrance, similar to those from China. It is suitable for physically strong people because it has strong medicinal properties. ***Yotei*-rhubarb**: *Yotei* grows abundantly along riversides and in rice fields. Its leaves, stems, and flowers are blue, and its fruits are as light as buckwheat. The common Japanese name is *shinoko*, also called *gishigishi* or *no*-rhubarb. ***Do*-rhubarb**: The stems, leaves, flowers, fruits, and roots all resemble the rhubarb cultivated in Japan, and those that are red in color are called *do*-rhubarb or *yama*-rhubarb. Its original name is *sambo,* and it is listed in the Compendium of Materia Medica (Li, 2004). The shape of the root is similar to that of rhubarb. |
| 1683 | Materia Medica of Decoctions and Precious Fragments (*Toeki Hengyoku Honzo*: 湯液片玉本草) (Takada, 1683) | **Rhubarb produced in Japan**: The rhubarb grown in Japan from seed coincides with the description of Compendium of Materia Medica (Li, 2004) in terms of the color of its stems, leaves, and roots. The rhubarb with the best fragrance has a strong effect. |
| 1697 | Complete Book of Agriculture (*Nogyo Zensho*: 農業全書) (Miyazaki, 1697) | ***Toh*-rhubarb**: Rhubarb is a crude drug sometimes used by physicians. It is a species of *toh*-rhubarb that grows in places such as Nagaike in Yamashiro area (part of the present Kyoto). The leaves are round and thick, similar to the leaves of *Farfugium japonicum*, with a slightly reddish stem. The root is slightly similar to *wa*-rhubarb, which came to Japan a long time ago, but is a different species. |
| 1698 | Complete Compendium of Materia Medica of Wide Benefit (*Koeki Honzo Taisei*:広益本草大成) (Okamoto, 1698) | ***Do*-rhubarb** (*Yama*-rhubarb): Its original name is *sambo*, and it is similar to rhubarb, but not a true species. |
| 1699 | Explanation and Commentary on Records of Medicinal Properties (*Yakushoki Benkai*: 薬性記弁解) (Okamoto, 1699) | ***Yotei*-rhubarb**: The plant with long, slender leaves is mistakenly called rhubarb, but its actual name is *yotei*. This is why *yotei* is commonly called rhubarb. It is also called *gishigishi*. *Yotei* is less potent than rhubarb. Rhubarb is characterized by its round leaves, which distinguish it from *yotei*. Rhubarb is very bitter in taste and has the strongest cooling property. |
| 1702 | Complete Book of Drug Processing (*Hosha Zensho*: 炮炙全書) (Ino, 1702) | **Rhubarb produced in Japan**: Rhubarb grows in Yamato (the present Nara), Yamashiro, and Tango (part of the present Kyoto). ***Yotei*-rhubarb**: Most of the rhubarb sold in stores is derived from *yotei* root and should not be counterfeited. |
| 1712 | Materia Medica Harmony and Interpretation (*Honzo Wage*: 本草和解) (Masatsugu, 1712) | **Rhubarb produced in Japan**: Rhubarb grown in Japan should not be used. Its stem, leaves, and roots do not coincide with the description in Compendium of Materia Medica (Li, 2004). The roots are similar to Chinese rhubarb and have the best fragrance. It is suitable for physically strong people because it has strong medicinal properties. |
| 1726 | Essential Knowledge for Using Medicines (*Yoyaku Suchi*: 用薬須知) (Matsuoka, 1726) | ***Do*-rhubarb**: There is a larger-leaved species called *do*-rhubarb, which has the same effects as *yotei*. |
| 1727 | Medicine Basket Materia Medica (*Yakuro Honzo*: 薬籠本草) (Katsuki, 1727) | ***Yotei*-rhubarb**: Most of Rhubarb sold in stores is derived from *yotei* root and should not be counterfeited or used. |
| 1738 | Ippondo's Selection of Crude Drugs (*Ippondo Yakusen*: 一本堂薬選) (Kagawa, 1738) | ***Yotei*-rhubarb**: It is incorrect to call the root of *yotei* (*gishigishi*) to *no*-rhubarb. ***Do*-rhubarb**: *Do*-rhubarb has shorter, rounder, larger leaves than *yotei*, similar to the leaves of rhubarb, but the edges are not chipped and the color is not the same. The root is poor quality and should not be used. |
| 1754 | Clarifying Confusions about Crude Drugs (*Yakuhin Benwaku*: 薬品弁惑) (Oguchi, 1754) | ***Shin*-rhubarb**: *Shin*-rhubarb comes from Tang and is grown in Yamashiro, Yamato, Tango, and so on. It has a brocade pattern. Although imported rhubarb has better quality, it is acceptable to use it if imported Chinese rhubarb is in short supply. |
| 1772 | Discrimination and Clarification of Materia Medica (*Honzo Benmei*: 本草辨明) (Hayashi, 1772) | **Rhubarb produced in Japan**: The color of stems, leaves, and roots of rhubarb grown in Japan differs from that of Chinese. It is suitable for physically strong people because it has the best fragrance and strong medicinal properties. |
| 1780 | Minimumal 6x8 Materia Medica (*Hengyoku Rokuhati Honzo*: 片玉六八本草) (Kato, 1780) | ***Wa*-rhubarb**: *Wa*-rhubarb is *gishigishi*. |
| 1790 | Pocket Compendium of Essential Materia Medica (*Syuchin Honzo Shun*: 袖珍本草雋) (Hirazumi, 1790) | **Rhubarb produced in Japan**: Rhubarb grown in Yamato is better at drugstores. Rhubarb grows in Yamato (the present Nara), Yamashiro and Tango (part of the present Kyoto). It is better to have a purple-colored brocade pattern. ***Yotei*-rhubarb**: Most of the Rhubarb sold is derived from *yotei* root and should not be counterfeited. |
| 1793 | Brief Compendium of Medicinal Herbs (*Yakuso Rhakuhu*: 薬草略譜) (Kyakuika, 1793) | ***Yotei*-rhubarb**: *Inu*-rhubarb, which has long leaves and grows wild in many suburban fields, is called *yotei* in Chinese. ***Toh*-rhubarb**: The Chinese name for *toh*-rhubarb is *sambo*, and the leaves are not prickly and slightly rounded. It grows in the mountains. |
| 1795 | Explanations of Crude Drugs in Classical Prescriptions (*Koho Yakusetsu*: 古方薬説) (Ujita, 1795) | **Rhubarb produced in Japan**: Seeds from China were transferred to various parts of Japan for cultivation and were found to be easy to grow. ***Do*-rhubarb**: *Do*-Rhubarb is sold in stores as the *Wa*-Rhubarb, but the *Do*-Rhubarb should not be used for medicinal purposes. However, *Do*-Rhubarb is effective in the treatment of skin infections. *Do*-Rhubarb grows abundantly along valley streams. Its seedlings, leaves, flowers, and fruits resemble those of *yotei*, but are larger. ***Toh*-rhubarb**: *Do*-Rhubarb is mistakenly called *Toh*-Rhubarb. ***Shin*-rhubarb**: The leaves are similar to those of *do*-rhubarb, broad and large, the stems are 1.8–2.1 m long. The roots are like thick arms with a purple brocade pattern. It is called *shin*-rhubarb. It can be sliced into rings and dried by tying them with a rope or cut into chunks that resemble an ox's tongue. |
| 1798 | Compilation and Commentary on the Compendium of Materia Medica (*Honzo Komoku Sanso*: 本草綱目纂疏) (So, 1798) | ***Do*-rhubarb**: Rhubarb that grows in the wild is *do*-rhubarb, not *shin*-rhubarb. ***Shin*-rhubarb**: Recently, a Chinese species of rhubarb has been grown in Yamato, Uda, and other areas. Stores call this species "*Shin*-Rhubarb." Due to its mild effect, it is not used in emergency situations. |
| 1805 | Elucidation of Compendium of Materia Medica (*Honzo Komoku Keimo*: 本草綱目啓蒙) (Ono, 1805) | ***Do*-rhubarb**: Shizhen was incorrect in stating that *sambo* is *do*-rhubarb. *Sambo* is known as *suiba* in Japanese and can be found in the aquatic plants section. *Karasunoabura* grows up to 1.5 m tall and has long leaves similar to burdock leaves. Its flowers and fruits resemble *yotei*, and its yellow roots grow in clusters. ***Shin*-rhubarb**: A Chinese species of rhubarb is imported and grown in Zhoshu (Yamashiro), Nagaike, and Washu (Yamato) and is called *Shin*-Rhubarb in stores. Because the cut end has a purple stripe, and this is a Rhubarb with a brocade pattern. The shape and color are the same as the imported ones. However, they are not as good as the imported rhubarb because they have not adapted to the Japanese soil. Some say that, after two years, their quality will be the same as Chinese rhubarb. The leaves are large and broad, similar to those of *Pterospermum acerifolium*, without serrations, glossy, and about 60 cm in length. |
| 1810 | Study of Crude Drugs for Daily Use (*Nichiyo Yakuhin Ko*: 日用薬品考) (Shibata, 1810) | ***Wa*-rhubarb**: Many of the items sold as *Wa*-Rhubarb are *Do*-Rhubarb, or the mixture of *Shin*-Rhubarb shavings and *Do*-Rhubarb. *Yotei* root (*no*-rhubarb) may be regarded as a low-quality *wa*-rhubarb. However, both these *do*-rhubarb and *yotei*-rhubarb should not be used as common rhubarb. ***Yotei*-rhubarb**: *Yotei* was considered rhubarb in Japan in the past, so the rhubarb in the old prescription used *yotei* root. ***Shin*-rhubarb**: In Japan, a Chinese species of rhubarb is grown, which is produced in large quantities in Washu and Uda, and is sold in drugstores. This is called *Shin*-Rhubarb, and the one with purple stripes on the cut end of the root is brocade-patterned Rhubarb. However, its properties and taste are inferior to imported Rhubarb.  The Rhubarb called "Korean-Rhubarb" in stores is this one. |
| 1823 | Augmented Edition of the Primer on the Palm (*Zoho Shuhan Hatsumo*: 増補手板発蒙) (Fujii, 1823) | ***Wa*-Rhubarb**: The Rhubarb now sold in drugstores as *Wa*-Rhubarb is *Do*-Rhubarb. It is similar to the root derived from *yotei*, but is a different species. ***Yotei*-rhubarb**: *Yotei* (*gishigishi*). ***Do*-rhubarb**: *Karasunoabura.* ***Shin*-rhubarb**: It comes mainly from the southern part of Japan. This is one of the Chinese species, but its effect is weak and it should not be used. |
| 1824 | Illustrated Sino-Japanese Encyclopedia of the Three Realms (*Wakan Sansai Zue*: 和漢三才図会) (Terashima, 1824) | ***Wa*-Rhubarb**: Now the *Wa*-Rhubarb is all derived from *yotei* or *suiba* with large roots. *Wa*-Rhubarb is effective in the treatment of some skin infections. ***Toh*-Rhubarb**: *Toh*-Rhubarb is used for its effect on bowel movements. |
| 1826 | Secret Formulas of Materia Medica (*Honzo Hiketsu*: 本草秘訣) (Unknown author, 1826) | **Rhubarb produced in Japan**: Four varieties of Rhubarb are produced in Japan. The first is the Chinese type, which is cultivated in Yamato, Yamashiro, and Tango. It is called "brocade patterned-Rhubarb." The second variety is *yotei*-rhubarb, also known as *gishigishi*. The third variety is called *do*-rhubarb. It is also called *ushinosita*, and its leaf is similar to that of *Nicotiana tabacum*. The Chinese name is unknown. The fourth variety is called *sambo*; its Japanese name is *suiba*. It was described that *sambo* is similar to *Ricinus communis*, but the truth is unclear. |
| 1827 | Illustrated Explanation of Poisonous Plants (*Yudoku Somoku Zusetsu*: 有毒草木図説) (Kiyohara, 1827) | **Rhubarb produced in Japan**: There is no Japanese indigenous rhubarb, but Chinese rhubarb has been transferred and cultivated. Since it is difficult to propagate, it is better to plant its roots. |
| 1830 | Newly Revised and Augmented Dutch Mirror of Medicine (*Shintei Zoho Oranda Yakkyo*: 新訂増補和蘭薬鏡) (Udagawa, 1830) | ***Do*-rhubarb**: Unscrupulous merchants sell *Do*-Rhubarb mixed with *yotei* root. |
| 1830 | Illustrated Manual of Materia Medica (*Honzo Zufu*: 本草図譜) (Iwasaki, 1830) | **Rhubarb produced in Japan**: Chinese rhubarb has been cultivated in many places after it was imported during the Kyoho era (1716–1736). ***Do*-rhubarb**: A rhubarb of *do*-rhubarb (Korean rhubarb, Shinshu rhubarb, *karasunoabura*) is produced in Shinshu area. It grows from planted roots in the spring and has large leaves, 12–15 cm wide and 30 cm long, that are slightly similar to *sambo*. Its stems are red, and its flowers and fruits resemble those of *sambo*. Its long, reddish-yellow roots resemble *toh*-rhubarb, and produce a yellow juice. It tastes very bitter and slightly spicy. It also has a purgative effect. |
| 1837 | Inquisitive Materia Medica (*Shitsumon Honzo*: 質問本草) (Go, 1837) | ***Do*-rhubarb**: *Do*-rhubarb has the following properties: cooling effects, the ability to purge poison from the body, and use throughout the body. |
| 1840 | Investigation of Crude Drugs in Classical Prescriptions (*Koho Yakuhin Ko*: 古方薬品考) (Naito, 1840) | **Rhubarb produced in Japan**: Chinese rhubarb has been cultivated in Washu area (the present Nara), which was imported during the Kyoho era. It differs from Chinese products since it does not grow in Japanese soil. ***Yotei*-rhubarb**: *Yotei*-rhubarb, also known as *gishigishi,* has long leaves and yellow roots. It should only be used externally. ***Shin*-rhubarb**: *Do*-rhubarb (*karasunoabura*) is similar in morphology to the Chinese species, but has slightly narrower leaves. This is called *shin*-rhubarb. It should only be used externally. |
| 1856 | Illustrated Explanation of Plants (*Somoku Zusetsu*: 草木図説) (Iinuma, 1856) | ***Do*-rhubarb** (*Karasunoabura*): *Do*-Rhubarb is used in place of Rhubarb at double the amount, as it has a laxative effect when planted in dry land. |
| 1859 | Study of Japanese Rhubarb (*Nihon Daioko*: 日本大黄考) (Shimizu, 1859) | ***Wa*-rhubarb**: *Wa*-rhubarb is called *ohoshi* and is different from Chinese rhubarb. |
| Unknown | Recorded Hearings on the Compendium of Materia Medica (*Honzo Komoku Kibun*: 本草綱目紀聞) written by Toyobumi Mizutani (Mizutani, unknown year) | **Rhubarb produced in Japan**: The Rhubarb produced in Japan is called Korean Rhubarb. It is the same as the Rhubarb imported from Korea in the past. Although it is similar to Chinese Rhubarb, it is called Korean Rhubarb for this reason. ***Yotei*-rhubarb**: *Yotei*, sometimes called *ohoshi*, is similar to *sambo* but larger. |
| Unknown | Recorded Hearings on Materia Medica (*Honzo Kibun*: 本草紀聞) written by Ranzan Ono (Ono, unknown year) | ***Toh*-rhubarb**: *Toh*-rhubarb introduced during the Kyoho era was cultivated in Yamashiro, Nagaike, and Sakabe. ***Shin*-rhubarb**: Many Japanese varieties are available at drugstores. The stores call them *“Shin*-Rhubarb”, which is produced by cutting the root and drying them. The roots sometimes have a brocade pattern like Chinese rhubarb. |

**SUPPLEMENTARY TABLE 2** Description of Rhubarb imported from China during the Edo period (1603–1868).

| **Year** | **Literature title** | ***Sogi-*Rhubarb** | ***Tsunagi-* (*Sengan-*) Rhubarb** | **Quality** |
| --- | --- | --- | --- | --- |
| 1680 | Illustrated Materia Medica (*Zukai Honzo*: 図解本草) (Shimotsu, 1680) | Nowadays, there is a product called *Sogi*-Rhubarb in drugstores. It is a type of *Shoku*-Rhubarb. A higher-quality product is firm, heavy, and moist. | *Tsunagi*-Rhubarb is slightly lighter and less moist. This is considered to be the second grade. | *Sogi* > *Tsunagi* |
| 1681 | Correction of Misunderstandings about Materia Medica (*Honzo Bengi*: 本草弁疑) (Endo, 1681) | *Sogi*-Rhubarb is a product made by cutting the Rhubarb at an angle and then drying it, and it is of higher quality because it is firm, heavy and moist. | *Tsunagi-*Rhubarb is a low-quality product made by threading Rhubarb through a rope and drying it like a string of pearls. It is light and should not be used. | *Sogi* > *Tsunagi* |
| 1683 | Materia Medica of Decoctions and Precious Fragments (*Toeki Hengyoku Honzo*: 湯液片玉本草) (Takada, 1683) | *Sogi*-Rhubarb, a product sold in drugstores, is cut at an angle and then dried. | *Tsunagi*-Rhubarb is dried like a string of pearls, and is of the lowest quality. | *Sogi* > *Tsunagi* |
| 1698 | Complete Compendium of Materia Medica of Wide Benefit (*Koeki Honzo Taisei*:広益本草大成) (Okamoto, 1698) | The cut Rhubarb sold in drugstores is called *Sogi*-Rhubarb. It is of higher quality because it is firm and heavy with a yellow color. | *Tsunagi*-Rhubarb has holes and is linked. It is considered low quality because it is light. | *Sogi* > *Tsunagi* |
| 1698 | Augmented Edition of the Lingbao Medicinal Properties, Efficiency, and Toxicity (*Zoho Reiho Yakusho Nodoku*: 増補霊寳薬性能毒) (Manase, 1698) |  | *Tsuri*-Rhubarb is the same as *Tsunagi*-Rhubarb. It is a high-quality product that is dried after being tied to a rope. | *Tsunagi > Sogi* |
| 1702 | Complete Book of Drug Processing (*Hosha Zensho*: 炮炙全書) (Ino, 1702) | The cut product is moist, firm, and heavy. | The product connected by a hole is light weight and lacks moisture. | *Sogi* > *Tsunagi* |
| 1710 | Record of Newly Formulated Preparations of Medicinal Varieties (*Yakushu Shin Seizaiki*: 薬種新製剤記) (Okunishi, 1710) | *Sogi-*Rhubarb is a high-quality product that is cut at an angle and then dried. | *Tsunagi-*Rhubarb is threaded onto a rope and dried like a string of pearls. It is considered the lowest quality due to its poor color, light weight, and lack of moisture. | *Sogi* > *Tsunagi* |
| 1712 | Materia Medica Harmony and Interpretation (*Honzo Wage*: 本草和解) (Masatsugu, 1712) | *Sogi*-Rhubarb is a product that is cut at an angle and then dried. It is high quality because it is firm, heavy, and moist. | *Tsunagi-*Rhubarb is threaded onto a rope and dried like pearls. It is the lowest quality because it is light and lacks moisture. | *Sogi* > *Tsunagi* |
| 1726 | Essential Knowledge for Using Medicines (*Yoyaku Suchi*: 用薬須知) (Matsuoka, 1726) | *Sogi* is not true Rhubarb. It is *yotei*. The Japanese name is *gishigishi*. It is also called *ushinoshita*, which means "cow's tongue." | *Tsunagi* is true Rhubarb, it is *Sengan*-Rhubarb in the ancient medicinal literature. | *Tsunagi > Sogi* |
| 1727 | Medicine Basket Materia Medica (*Yakuro Honzo*: 薬籠本草) (Katsuki, 1727) | The cut product is cow's tongue Rhubarb in ancient herbal literature. | The fist-like lump was introduced in modern times from the Qing Dynasty. The highest quality has a purple brocade pattern on the cut edge. This type of Rhubarb is referred to as "*sengan*" in ancient medicinal literature. | *Tsunagi > Sogi* |
| 1738 | Ippondo's Selection of Crude Drugs (*Ippondo Yakusen*: 一本堂薬選) (Kagawa, 1738) | The cut product like cow's tongue is made in Japan, while the brocade patterned one is made in China. | The products connected by a hole have higher quality. | *Tsunagi > Sogi* |
| 1772 | Discrimination and Clarification of Materia Medica (*Honzo Benmei*: 本草辨明) (Hayashi, 1772) | *Sogi*-Rhubarb is a high-quality product that is cut at an angle and dried. It is firm, heavy, and moist. | *Tsunagi*-Rhubarb is a product threaded through a rope and dried like a string of pearls, and is of the lowest quality because it is light and should not be used. | *Sogi* > *Tsunagi* |
| 1780 | Minimumal 6x8 Materia Medica (*Hengyoku Rokuhati Honzo*: 片玉六八本草) (Kato, 1780) | *Sogi*-Rhubarb, a product imported from Continental China, is referred to as "cow's tongue Rhubarb" in ancient medicinal literature. This Rhubarb is derived from *gishigishi* and should not be used. It is incorrect to say that *Sogi*-Rhubarb is used. | *Tsunagi*-Rhubarb imported from Continental China should be used, which is called *Sengan*-Rhubarb in the ancient medicinal literature. | *Tsunagi > Sogi* |
| 1790 | Pocket Compendium of Essential Materia Medica (*Syuchin Honzo Shun*: 袖珍本草雋) (Hirazumi, 1790) | The cut product is not true Rhubarb; it is the root of *yotei*. | The product called *Tsunagi* (*Sengan*) that was introduced by Zhoan Matsuoka is true Rhubarb. | *Tsunagi > Sogi* |
| 1795 | Explanations of Crude Drugs in Classical Prescriptions (*Koho Yakusetsu*: 古方薬説) (Ujita, 1795) |  | A purple, brocade-like, bitter-tasting clump from Continental China that has holes in it and is connected is called *Tsunagi*-Rhubarb. | Only *Tsunagi* |
| 1798 | Compilation and Commentary on the Compendium of Materia Medica (*Honzo Komoku Sanso*: 本草綱目纂疏) (So, 1798) |  | Some of the imported products have holes in them and are connected. |  |
| 1805 | Elucidation of Compendium of Materia Medica (*Honzo Komoku Keimo*: 本草綱目啓蒙) (Ono, 1805) | *Sogi*-Rhubarb, a product sold in drugstores, is cut at an angle and then dried. It used to be imported, but it is no longer imported. It is the cow's tongue described in the medicinal book of Zhangqi Chen (687?–757). | The *Tsunagi*-Rhubarb sold in drugstores is cut into thin, lengthwise slices, threaded onto a rope, and dried. It is the product introduced by Gong Su (599–674), who called *sengan*. |  |
| 1810 | Study of Crude Drugs for Daily Use (*Nichiyo Yakuhin Ko*: 日用薬品考) (Shibata, 1810) | *Sogi*-Rhubarb, a product sold in drugstores, is cut at an angle and then dried. It is the cow's tongue described in the medicinal book of Zhangqi Chen (687?–757). | *Tsunagi*-Rhubarb, also known as *Tsurushi*-Rhubarb, is cut into thin slices lengthwise, threaded onto a tree branch or rope, and dried. It is the product introduced by Gong Su (599–674), who called *sengan*. | Only *tsunagi* |
| 1823 | Augmented Edition of the Primer on the Palm (*Zoho Shuhan Hatsumo*: 増補手板発蒙) (Fujii, 1823) | According to the Dutchman's theory, the cow's tongue pieces are from the mountain district in Continental China. | *Sengan*-Rhubarb is produced in Russia. |  |
| 1824 | Illustrated Sino-Japanese Encyclopedia of the Three Realms (*Wakan Sansai Zue*: 和漢三才図会) (Terashima, 1824) | *Sogi*-Rhubarb is a sliced product that has been cut lengthwise or crosswise. | *Tsunagi*-Rhubarb is a product threaded through a rope and dried. |  |
| 1830 | Newly Revised and Augmented Dutch Mirror of Medicine (*Shintei Zoho Oranda Yakkyo*: 新訂増補和蘭薬鏡) (Udagawa, 1830) |  | The product connected by a hole is called Russian Rhubarb in the drugstores. |  |
| 1840 | Investigation of Crude Drugs in Classical Prescriptions (*Koho Yakuhin Ko*: 古方薬品考) (Naito, 1840) | The slices with cow's tongue are no longer imported. | *Sengan*-Rhubarb has higher quality. | *Tsunagi > Sogi* |
| Un-known | Mirror of Materia Medica (*Honzo Kagami*: 本草鏡) (Saito, unknown year) | S*ogi*-Rhubarb is not true product, it is the roots of *gishigishi*. |  |  |

**SUPPLEMENTARY TABLE 3** Descriptions of Rhubarb in Japanese literature during the Meiji era (1868–1912).

| **Year** | **Literature title** | **Descriptions of Rhubarb** |
| --- | --- | --- |
| 1878 | Recorded Hearings of Daily Lectures on Materia Medica (薬物学 日講紀聞) (Ermerins, 1878) | **Chinese Rhubarb**: Produced in China. **Russian Rhubarb**: It is produced in China and has the best quality. |
| 1878 | Outline of Materia Medica (薬物学大意) (Ota, 1878) | **Chinese, Russian, and European Rhubarb**: Sold in drugstores. |
| 1888 | Pharmacognosy (生薬学) (Wigand, 1888) | **Chinese Rhubarb**: The plant name is *Rheum officinale*, which is produced in continental China. Chinese Rhubarb is different from the product commonly called *Toh*-Rhubarb in Japan. **European Rhubarb**: [Plant name] *Rheum rhabarbarum*, *Rheum palmatum*, *Rheum compactum*, *Rheum emodi*. [Produced area] England, France, Australia, and Japan, etc. |
| 1889 | Practical Methods for Medicine Identification (実用薬品鑑定法) (Sano, 1889) | **Chinese Rhubarb**: The Rhubarb is mostly produced in continental China. Rhubarb derived from the root of Asian variety is of the highest quality and adapts to the criteria of the Japanese Pharmacopoeia. **European Rhubarb**: The quality of European Rhubarb roots is lower than that of Chinese rhubarb roots. |
| 1891 | Supplement to the Japanese Pharmacopoeia Revised Edition (改正日本薬局方随伴) (Kashimura and Ise, 1891) | **Chinese Rhubarb**: The original plants of Rhubarb have not been elucidated. We believed in the past the original plant was *Rheum palmatum*, but now *Rheum officinale* is referenced as the main original plant.  **European Rhubarb**: European or British Rhubarb is available on the market, but it should not be used as Rhubarb. ***Toh*-Rhubarb**: The Rhubarb used by traditional Kampo physicians is called *Toh*-Rhubarb. It is derived from a different type of *Rheum* plant that is typically rotten and softened. The characteristic actinocyte and medullary patterns of Rhubarb are unknown. |
| 1891 | Commentary on the Japanese Pharmacopoeia (日本薬局方註釈) (Sugiyama and Suda, 1891) | **Chinese Rhubarb**: Rhubarb is the rhizome of *Rheum* plants (Polygonaceae). However, the exact number of species of the original plant is not yet known. The most important species are *Rheum officinale*, which grows in southeastern Tibet and eastern Inner Mongolia in northwestern China, and *Rheum palmatum*, which grows in northeastern Tibet. **European Rhubarb**: European or British Rhubarb is derived from the roots of *Rheum* plants, which is grown in Europe. These kinds of Rhubarb have completely different qualities from the real substances and should not be used as substitutes. The original plants of these Rhubarb are not yet known. ***Toh*-Rhubarb**: The Rhubarb sold in drugstores in Japan as *Toh*-Rhubarb is usually rotten and softened. The characteristic actinocyte and medullary patterns of Rhubarb are not present. |
| 1896 | The Botanical Magazine (植物学雑誌) (Makino, 1896) | ***Do*-Rhubarb**: *Rumex madaio* Makino is *madaio* (*do*-rhubarb). |
| 1897 | General Flora of Great Japan (大日本普通植物誌) (Saita, 1897) | ***Toh*-rhubarb**: It is also called *ohoshi*, i.e., *Rheum rhabarbarum* L.: The leaves are wavy, and the upper surface of the petiole is flat. [Effect] It is one of the most famous medicinal plants. ***Do*-rhubarb**: *Madaio* (*Rumex aquaticus* L. var. *japonicus* Meisn.) |
| 1897 | Preface to the Complete Works of Yukichi Fukuzawa (福澤全集緒言) (Fukuzawa, 1897) | ***Wa*-Rhubarb**: Seven or eight years ago, when there were no imports from continental China, the price of Rhubarb rose so high that poor and sick people could not afford it. They substituted a Japanese product called *Wa*-Rhubarb, which was ineffective and caused stomach aches. In recent years, Rhubarb has become more plentiful and less expensive due to increased trade. As a result, even poor people no longer suffer from stomachaches after taking *Wa*-Rhubarb. |
| 1904 | Pharmacognosy (生薬学) (Shimoyama, 1904) | **Chinese Rhubarb**: Though the original plants of Rhubarb have not been completely cleared, two species of the Polygonaceae family, *Rheum officinale* Baill. and *Rheum palmatum* L., which are grown in central China, especially on the mountain ridges near the Yellow River, are recognized as the original plants.  **European Rhubarb**: Rhubarb imported from European countries, including British, Austrian, and French varieties, and others. These kinds of Rhubarb are not suitable for medical or medicinal use due to their low quality. These kinds of Rhubarb are derived from the plants such as *Rheum rhabarbarum*, *Rheum rhaponticum*, and *Rheum compactum.*  ***Toh*-Rhubarb**: Traditional Kampo physicians used this type of Rhubarb called *Toh*-Rhubarb. It is typically rotten and softened, and its characteristic actinocyte and medullary patterns are unknown. It is derived from different species as Chinese Rhubarb. **Rhubarb produced in Japan**: *Tsugaru*-Rhubarb is the highest quality Rhubarb produced in Japan. Its shape is similar to Austrian Rhubarb, and it may be the roots of *Rheum rhabarbarum* L. |
| 1909 | Concise Pharmacognosy (簡明生薬学) (Shimazaki, 1909) | **European Rhubarb**: European Rhubarb is derived from the roots of *Rheum* plants. They are long and slender, and unlike Chinese Rhubarb, they do not have a brocade pattern. They are also of low quality for medicinal use. Therefore, the two should not be confused. Austrian and Hungarian Rhubarb are available as substitutes for Chinese Rhubarb. These are derived from species such as *Rheum undulatum*, *Rheum rhaponticum*, and *Rheum compactum*. ***Toh*-Rhubarb**: *Toh*-Rhubarb is rotten and softened, and the characteristic actinocyte and medullary pattern of Rhubarb are unknown. **Rhubarb produced in Japan**: Rhubarb produced in Tsugaru has the highest quality. The shape of this type of Rhubarb is similar to that of Austrian Rhubarb, and it may be the roots of *Rheum undulatum* L. ***Wa*-Rhubarb**: *Wa*-Rhubarb produced in Japan is the roots of *Rumex japonicum* and *Rumex aquaticus*, etc., and those are different from *Rheum* plant. |
| 1910 | Comprehensive Pharmacology (集成薬物学) (Ise, 1910) | **Chinese Rhubarb**: Rhubarb is the dried and peeled giant rhizome of *Rheum officinalis*, *Rheum palmatum*, and other *Rheum* species, family Polygonaceae, grown in high altitudes in the central or northern part of China. **European Rhubarb**: The roots of European Rhubarb, such as *Rheum rhaponticum*, are long and slender. Unlike Chinese Rhubarb, they do not have a brocade pattern and are of poor quality for medicinal use. Therefore, the two should not be confused. ***Wa*-Rhubarb**: *Wa*-Rhubarb produced in Japan is the roots of the *Rumex japonicum* and *Rumex aquaticus*. These are different from the *Rheum* plant but belong to the same family. |
| 1910 | Study of Wakanyaku (和漢薬考) (Koizumi, 1910) | **Chinese Rhubarb**: The product derived from *Rheum officinale*, *Rheum rhabarbarum*, *Rheum. spiciforme*, *Rheum emoodi*, etc. **European Rhubarb**: The product derived from *Rheum rhabarbarum*, *Rheum palmatum*, *Rheum compactum*, *Rheum hydridum*, etc. |
| 1911 | Pocket Pharmacognosy (ポケット生薬学) (Nishimura, 1911) | **European Rhubarb**: European Rhubarb, e.g., British, French, and Austrian, is of low quality and not used for medicinal purposes. Its original plants are *Rheum emodi* Wall., *Rheum rhabarbarum* L., *Rheum compactum* L., *Rheum rhaponticum* L., etc. ***Wa*-Rhubarb**: Its original plant is grown in Japan, and is the root of *Rheum rhabarbarum* L. |
| 1911 | Essentials of Pharmacognosy (生薬学要訣) (Nakajima, 1911) | **Chinese Rhubarb**: The Japanese Pharmacopoeia registers only Rhubarb derived from continental China. **European Rhubarb**: British Rhubarb is the rhizome of *Rheum rhabarbarum*; Austrian Rhubarb is the rhizome of *Rheum rhaponticum;* French Rhubarb is the rhizome of *Rheum compactum*. ***Toh*-Rhubarb**: *Toh*-Rhubarb is usually rotten and softened, and should not be used for medicine. **Rhubarb produced in Japan**: Japanese Rhubarb might be the same as British Rhubarb. |

**SUPPLEMENTARY TABLE 4** Descriptions of Rhubarb in Japanese literature during the Taisho era (1912–1926).

| **Year** | **Literature title** | **Descriptions of Rhubarb** |
| --- | --- | --- |
| 1913 | Complete Compendium of Modern Pharmacy (最新薬学全書) (Ito, 1913) | ***Toh*-Rhubarb**: It was used by Kampo physicians in earlier days. It is usually rotten and softened. **Rhubarb produced in Japan**: It is the root of *Rheum rhabarbarum,* and its best quality is produced in Tsugaru. |
| 1916 | Cultivation and Study of Medicinal Plants (薬草栽培と其研究) (Kanan and Kamekawa, 1916) | **Rhubarb produced in Japan**: It is derived from *Rheum rhabarbarum*, and that of produced in Tsugaru is praised. |
| 1918 | Cultivation and Marketing of Important Medicinal Plants (重要薬草栽培と其販売法) (Bandai, 1918) | ***Toh*-Rhubarb**: In Japan, Tsugaru-gun, Aomori Prefecture, and Kuhonai, Kudo-gun, Iwate Prefecture, produce excellent products. It is called *Toh*-Rhubarb and has been an important medicine for Kampo physicians since ancient times. |
| 1918 | Annotated Complete Collection for Pharmacist Examination Questions and Answers (説明附薬剤師試験問題答案全集) (Onda, 1918) | **European Rhubarb**: The cultivated rhubarb in British, Austria, and French are *Rheum rhabarbarum*, *Rheum rhaponticum*, and *Rheum. compactum*. The active ingredients of anthraquinone derivatives are less contained. ***Toh*-Rhubarb**: The original plant of *Toh*-Rhubarb might be derived from *Rheum rhabarbarum*. Since Kampo physicians praise the Rhubarb, it is also called general. However, many of them are so corroded that their typical structure is not visible.  **Rhubarb produced in Japan**: *Wa*-Rhubarb, might be derived from *Rumex daiwoo* Makino (*Shin*-Rhubarb). ***Wa*-Rhubarb**: Same as the Rhubarb produced in Japan. |
| 1919 | Great Survey of Japanese Medicinal Plants: Cultivation and Harvesting (日本薬草大観栽培採収) (Kawamura, 1919) | ***Toh*-Rhubarb**: Same as the Rhubarb produced in Japan. **Rhubarb produced in Japan**: The Rhubarb produced in Japan is called *Toh*-Rhubarb, and the highest quality is produced in the Aomori Prefecture. The Rhubarb is similar to that produced in Europe. Its root is cylindrical. They reportedly have similar medicinal properties to those of European Rhubarb. Therefore, even the best Japanese products are not as good as Chinese products. |
| 1920 | The Japanese Pharmacopoeia Fourth Edition (Choyokai Co., 1920) | ***Toh*-Rhubarb**: *Toh*-Rhubarb (*Todaiwo*) is the dried rhizome of *Rheum* plants grown in continental China. |
| 1920 | Cultivation of Medicinal Plants for New Drugs (新薬植物栽培) (Matsuda, 1920) | **European rhubarb**: The rhubarb cultivated in England and Germany is *Rheum palmatum*. ***Toh*-rhubarb**: The original plant is *Rheum rhabarbarum,* its Japanese plant name is *karadaio.* It seems different from the Chinese products. **Rhubarb produced in Japan**: The Rhubarb produced in Japan is called *Wa*-Rhubarb, and may be derived from *Rheum rhabarbarum* L. The wild root is harvested in continental China, while Japan uses the cultivated root. ***Wa*-Rhubarb**: The most famous *Wa*-Rhubarb in Japan has been produced in Tsugaru since ancient times. Nara Prefecture is the main producing area in Japan, but there is not enough to meet domestic demand. Therefore, 90–96 tons are imported from China each year. |
| 1923 | Concise Lectures for Drug Merchants (簡明薬種商講義) (Maruyama, 1923) | ***Toh*-Rhubarb**: It is commonly called *karadaio* (its original plant name in Japan), which is the dried root of *Rheum rhabarbarum* produced in China. It also contains emodin, the same as Rhubarb. It is used as a substitute for Rhubarb as a laxative. It has been used by Kampo physicians, and it is said that the effect of laxatives is better than Rhubarb. |
| 1923 | Materia Medica for Drug Merchants (薬種商薬物学) (Koyama, 1923) | ***Toh*-Rhubarb**: It is the dried root of *Rheum* plants grown in China, which is newly listed in the 4th Japanese Pharmacopoeia, and it has been praised by Kampo physicians. It is used as a laxative and stomachic. |
| 1924 | The Japanese Pharmacopoeia Fourth Edition for Physicians with Prescriptions (処方添註医家用第四改正日本薬局方) (Nagao, 1924) | ***Toh*-Rhubarb**: It is the dried rhizome of *Rheum* plants produced in China. It has the same medicinal effects as rhubarb and is mainly used as a raw material for medicines. |
| 1925 | Detailed Explanations of New Medicines (新薬詳解) (Ryosaku, 1925) | ***Toh*-Rhubarb:** The medicinal effects of *Toh*-Rhubarb are the same as those of Rhubarb; it is mainly used as a raw material for medicines. The ingredients are the same as Rhubarb. |
| 1925 | Concise Lectures for Drug Merchants (薬業講習書) (Hino, 1925) | ***Toh*-Rhubarb** [Origin] The dried rhizome of *karadaio*, the Japanese name for *Rheum rhabarbarum*, that is produced in China. [Ingredients] It contains emodin. [Effect] The medicinal effects are the same as those of Rhubarb, but its purgative action is less severe than that of Rhubarb and is not accompanied by abdominal pain. |
| 1925 | Modern Medicines in Clinical Practice (臨床近世薬物) (Nagao, 1925) | ***Toh*-Rhubarb**: It is the dried root of *Rheum* plants produced in China. It is used as a substitute for Rhubarb. |

**SUPPLEMENTARY TABLE 5** Descriptions of Rhubarb in Japanese literature during the Showa era (1926–1989).

| **Year** | **Literature title** | **Descriptions of Rhubarb** |
| --- | --- | --- |
| 1928 | Register of Domestic Products (国産台帳) (Kokusan Shinkokai, 1928) | ***Toh*-Rhubarb**: Same as the *Wa*-Rhubarb. ***Wa*-Rhubarb**: The original plant of *Wa*-Rhubarb is slightly different from that of *Toh*-Rhubarb, but these two have similar medicinal effects, and these production prices are not competitive. |
| 1929 | Medicinal Plants for Home Knowledge (家庭須知薬用植物) (Kyushu Medicinal Plant Research Society, 1929) | ***Toh*-rhubarb**: *Karadaio* (the Japanese name for *Rheum rhabarbarum*), i.e., *Toh*-Rhubarb was first officially listed in the 4th Edition of the Japanese Pharmacopoeia. ***Do*-rhubarb**: *Shin*-rhubarb; polygonaceae family. There is a plant commonly called as *karasunoabura*. It is a perennial herb that grows in deep mountain valleys and wetlands near water. It has large leaves, about 30 cm long and 20 cm wide, and spike-shaped flowers similar to those of rhubarb. Its medicinal properties are similar to those of Rhubarb. |
| 1929 | Supplementary Notes on Kampo Medicines (漢方医学余談) (Nakayama, 1929) | ***Toh*-Rhubarb**: Westerners use Turkish Rhubarb as a laxative, but when Japanese use it, it causes severe abdominal pain, so Japanese must use *Toh*-Rhubarb. |
| 1932 | The Japanese Pharmacopoeia Fifth Edition (Yakugyo jiho Co., 1932) | ***Toh*-Rhubarb**: Rhizoma Todaiwo (Another name of *Toh*-Rhubarb) is dried rhizome of *Rheum* plants produced in China. |
| 1940 | Makino's illustrated flora of Japan (牧野日本植物図鑑) (Makino, 1940) | ***Toh*-rhubarb**: *Karadaio*, i.e., *Rheum rhabarbarum* L. It is a large perennial herb that was planted when it came to China in the Edo period, originally from the Siberia region. The Japanese name is *toh*-rhubarb, meaning "Chinese rhubarb," and in the Edo period it was mistaken for true rhubarb, i.e., *shin*-rhubarb, but it is not true rhubarb and has no medicinal value.  The *Engishiki* states that rhubarb, called *ohoshi* (meaning large *gishigishi*), was offered as tribute from various regions. "Shi" is the old name for *yotei* or *gishigishi*. However, this rhubarb may have been *shin*-rhubarb, which was produced in Japan. It is difficult to imagine that it was cultivated in many regions 1,000 years ago.  [Chinese name] Rhubarb (misuse). ***Do*-rhubarb**: *Madaio*; *Rumex daiwoo* Makino. The species name *daiwoo* is based on the fact that it was once mistaken for medicinal rhubarb. Its Japanese name means true rhubarb, but it was originally misidentified and is not actually a true rhubarb.  [Name] *Do*-rhubarb (misuse). |
| 1940 | New Edition of Medicinal Botany (薬用植物学) (Koizumi, 1940) | ***Toh*-rhubarb**: *Rheum rhabarbarum* (*wa*-rhubarb. *ohoshi*, *toh*-rhubarb)  [Distribution and form] Originally from northern China and Siberia, it was cultivated in China and imported to Japan in ancient times and planted in Nara and Tokushima prefectures. It is called *wa*-rhubarb. The leaves have long petioles, ovate in shape, with shallowly lobed and contracted margins. [Crude drug] *Toh*-Rhubarb (*wa*-Rhubarb): “Rhizoma Karadaiwo” is a peeled and dried rhizome with a yellowish-brown cut surface. It has a slightly bitter and astringent taste. [Ingredients]The rhizome contains glycoside decomposition products such as emodin, and the active ingredient is anthraquinone glycoside. [Medicinal effects] This product is a substitute for genuine Rhubarb and has the same effect. [Remarks] Introduced to Japan from China in 1875, and that of produced in Tsugaru has best quality. |
| 1941 | Studies on Japanese Medicines (国医薬物学研究) (Shimizu, 1941) | ***Toh*-Rhubarb**: It is dried rhizome of *Rheum* plants produced in China, which is described as “Rhizoma Todaiwo” in Japanese Pharmacopoeia. ***Wa*-Rhubarb**: This is the root of *Rheum rhabarbarum*, but nowadays many use the root of *Rumex daiwoo*, which is a fake *Toh*-Rhubarb. Be careful when buying cut products, as sometimes the root of *Rumex japonicus* is used. |
| 1943 | Pharmacognosy (生薬学) (Shimoyama, 1943) | ***Toh*-Rhubarb**: Rhizoma Todaiwo The product called *Toh*-Rhubarb is said to be derived from the Polygonaceae plant of *Rheum rhabarbarum* L., however this theory is not reliable. This was registered in the 4th Edition of the Japanese Pharmacopoeia because it is imported from China and used as a price by Kampo physicians. ***Wa*-Rhubarb**: It is produced in Japan. Rhubarb produced in Tsugaru has been famous since ancient times and is still cultivated in Nara Prefecture. The original plant is Polygonaceae family of *Rheum rhabarbarum* L. |
| 1947 | Latest Classified Compilation of Pharmaceutical Products (最新医薬品類聚) (Yoshimatsu, 1947) | ***Toh*-Rhubarb** [History] It was imported from China and used specifically by Kampo physicians, and was registered in the fourth edition of the Japanese Pharmacopoeia as the name of Todaiwo, then changed to Rhizoma Todaiwo in the fifth edition. [Crude drug] The Japanese Pharmacopoeia describes it as "the dried rhizome of *Rheum* plants grown in China” and the original plant is unknown. There is some suggestion that the original plant may have been *Rheum rhabarbarum*, but this is not certain. The ingredients are said to be a glycoside composed of anthraquinone derivatives such as emodin, but there are no exact reports. The ash content is lower than that of the regular Rhubarb derived from *Rheum palmatum* var. *tanguticum* (the synonym of *Rheum tanguticum*). Its medicinal effects are similar to those of Rhubarb, but its laxative effect is less severe than that of Rhubarb and it does not cause abdominal pain, so it is highly valued as a laxative in Japan. **Rhubarb produced in Japan**: Since ancient times, Tsugaru Rhubarb has been famous for its high quality, and even today it is more expensive than other Japanese Rhubarb (*Wa*-Rhubarb). *Wa*-Rhubarb is mainly produced in Nara and Tokushima prefectures. This Rhubarb is said to be derived from *Rheum rhabarbarum*, but it should not be confused with *Toh*-Rhubarb (*Todaiwo*), as they are different.  Emodin and chrysophanlic acid have been found as ingredients. It is used in substitutes for Rhubarb or *Toh*-Rhubarb as a laxative or stomachic, but its action as a laxative is the mildest. Rhubarb grown in Nara Prefecture has a strong laxative effect, but is said to cause abdominal pain.  In addition to the above, the roots of *Rumex japonicus* and *Rumex acetosa* are also treated as Rhubarb in Japan, but are said to cause severe abdominal pain. Tomitaro Makino also mentioned *do*-rhubarb (*Rumex daiwoo* Makino) as a kind of rhubarb. It is described: "When planted in dry land, it is effective in curing diarrhea, so double the amount of rhubarb is used to substitute for it. It also describes: "*No*-rhubarb (*Rumex domesticus*) is abundant in the northern part of Japan. *Ma*-rhubarb, *ma* is another reading of the same Kanji as *shin*, is abundant in the southern part (author's note: not the southern part of the Tsugaru region.)" *Ma*-Rhubarb means true Rhubarb, and in the past, it has been mistaken for Rhubarb. |
| 1951 | The Japanese Pharmacopoeia Sixth Edition (Japan Pharmaceutical Association, 1951) | ***Wa*-Rhubarb**: Rheum Japonicum; Rheum Jap. *Wa*-Rhubarb is derived from the short, columnar, upright rhizome and thick, columnar roots of *Rheum rhabarbarum* Linne (Polygonaceae), which are split transversely or longitudinally and then dried. |
| 1952 | llustrated Guide to Medicinal Plants (薬用植物図説) (Murakoshi, 1952) | ***Wa*-rhubarb**: *Rheum rhabarbarum* L.; *Ohoshi* It is a perennial herb native to Siberia that is cultivated in Japanese fields.  The crude drug derived from this species is *Wa*-Rhubarb. (Japanese Pharmacopoeia) Emodin and crysophanic acid are included in the root and rhizome. As a substitute for true Rhubarb, a small amount of "*Wa*-Rhubarb" is used to treat indigestion. A large amount is used as a laxative in the form of a powder or decoction, with a recommended daily dosage of 2 – 4 g.  This plant can be grown in both cold and warm climates, but it is best grown in warm areas with light, soft soil that is moist in places. *Wa*-Rhubarb is in high demand as a medicinal raw material and is widely grown in Nara, Tokushima, Kumamoto, and other regions. |
| 1961 | The Japanese Pharmacopoeia Seventh Edition Pharmacopoeia(Pharmaceutical and Medical Device Regulatory Science Society of Japan, 1961) | ***Wa*-rhubarb**: Rheum Japonicum; Rheum Jap. *Wa*-Rhubarb is derived from the short, columnar, upright rhizome and thick, columnar roots of *Rheum rhabarbarum* Linne (Polygonaceae), which are split transversely or longitudinally and then dried. |

**References**

Bandai, T. (1918). Cultivation and Marketing of Important Medicinal Plants (重要薬草栽培と其販売法). Tokyo: National Diet Library Digital Collections, 22–24. https://dl.ndl.go.jp/pid/931634

Choyokai Co. (1920). The Japanese Pharmacopoeia Fourth Edition. Tokyo: National Diet Library Digital Collections, 148. https://dl.ndl.go.jp/pid/931676

Endo, G. (1681). Correction of Misunderstandings about Materia Medica (*Honzo Bengi*: 本草弁疑). Tokyo: National Diet Library Digital Collections, 19. https://dl.ndl.go.jp/pid/2557651

Ermerins, J. (1878). Recorded Hearings of Daily Lectures on Materia Medica (薬物学 日講紀聞), transrated by Mise, M. Tokyo: National Diet Library Digital Collections, 14–16. https://dl.ndl.go.jp/pid/837744

Fujii, K. (1823). Augmented Edition of the Primer on the Palm (*Zoho Shuhan Hatsumo*: 増補手板発蒙). Kyoto: Kyoto University Rare Materials Digital Archive, 27. https://rmda.kulib.kyoto-u.ac.jp/item/rb00003037

Fukuzawa, Y. (1897). Preface to the Complete Works of Yukichi Fukuzawa (福澤全集緒言). Tokyo: National Diet Library Digital Collections, 18. https://dl.ndl.go.jp/pid/1083585

Go, K. (1837). Inquisitive Materia Medica (*Shitsumon Honzo*: 質問本草). Tokyo: National Diet Library Digital Collections, 34–35. https://dl.ndl.go.jp/pid/2556214

Hayashi, S. (1772). Discrimination and Clarification of Materia Medica (*Honzo Benmei*: 本草弁明). National Diet Library Digital Collections, 58–59. https://dl.ndl.go.jp/pid/2536779

Hino, G. (1925). Concise Lectures for Drug Merchants (薬業講習書). Tokyo: National Diet Library Digital Collections, 67–68. https://dl.ndl.go.jp/pid/922699

Hirazumi, S. (1790). Pocket Compendium of Essential Materia Medica (*Syuchin Honzo Shun*: 袖珍本草雋). Tokyo: National Diet Library Digital Collections, 122–124. https://dl.ndl.go.jp/pid/2536830

Iinuma, C. (1856). Illustrated Explanation of Plants (*Somoku Zusetsu*: 草木図説). Tokyo: National Diet Library Digital Collections, 33–36. https://dl.ndl.go.jp/pid/2558241

Ino, N. (1702). Complete Book of Drug Processing (*Hosha Zensho*: 炮炙全書). Tokyo: National Diet Library Digital Collections, 37. https://dl.ndl.go.jp/pid/2556035

Ise, J. (1910). Comprehensive Pharmacology (集成薬物学) Fifth Edition Tokyo: National Diet Library Digital Collections, 29–31. https://dl.ndl.go.jp/pid/1082206

Ito, H. (1913). Complete Compendium of Modern Pharmacy (最新薬学全書). Tokyo: National Diet Library Digital Collections, 30–31. https://dl.ndl.go.jp/pid/931567

Iwasaki, T. (1830). Illustrated Manual of Materia Medica (*Honzo Zufu*: 本草図譜). Tokyo: National Diet Library Digital Collections, 4–8. https://dl.ndl.go.jp/pid/1287131

Japan Pharmaceutical Association (1951). The Japanese Pharmacopoeia Sixth Edition. Tokyo: Japan Pharmaceutical Association, 369–370.

Kagawa, S. (1738). Ippondo's Selection of Crude Drugs (*Ippondo Yakusen*: 一本堂薬選). Tokyo: National Diet Library Digital Collections, 54–56. https://dl.ndl.go.jp/pid/2556221

Kanan, Y. and Kamekawa, K. (1916). Cultivation and Study of Medicinal Plants (薬草栽培と其研究). Tokyo: National Diet Library Digital Collections, 55. https://dl.ndl.go.jp/pid/931605

Kashimura, K. and Ise, J. (1891). Supplement to the Japanese pharmacopoeia Revised Edition (改正日本薬局方随伴). Tokyo: National Diet Library Digital Collections, 103–104. https://dl.ndl.go.jp/pid/837850

Kato, K. (1780). Minimumal 6x8 Materia Medica (*Hengyoku Rokuhachi Honzo*: 片玉六八本草). Tokyo: National Diet Library Digital Collections, 108–111. https://dl.ndl.go.jp/pid/2575936

Katsuki, G. (1727). Medicine Basket Materia Medica (*Yakuro Honzo*: 薬籠本草). Tokyo: National Diet Library Digital Collections, 38–41. https://dl.ndl.go.jp/pid/2557070

Kawamura, K. (1919). Great Survey of Japanese Medicinal Plants: Cultivation and Harvesting (日本薬草大観 栽培採収). Tokyo: National Diet Library Digital Collections, 138–140. https://dl.ndl.go.jp/pid/981127

Kiyohara, S. (1827). Illustrated Explanation of Poisonous Plants (*Yudoku Somoku Zusetsu*: 有毒草木図説). Tokyo: National Diet Library Digital Collections, 21. https://dl.ndl.go.jp/pid/2556216

Koizumi, E. (1910). Study of Wakanyaku (和漢薬考) Second Edition. Tokyo: National Diet Library Digital Collections, 186–189. https://dl.ndl.go.jp/pid/837911

Koizumi, H. (1940). New Edition of Medicinal Botany (新編薬用植物学) Tokyo: National Diet Library Digital Collections, 61–63. https://dl.ndl.go.jp/pid/1057209

Kokusan Shinkokai (1928). Register of Domestic Products (国産台帳). Tokyo: National Diet Library Digital Collections, 152–153. https://dl.ndl.go.jp/pid/1188697

Koyama, Y. (1923). Materia Medica for Drug Merchants (薬種商薬物学). Tokyo: National Diet Library Digital Collections, 156. https://dl.ndl.go.jp/pid/919195

Kyakuika, S. (1793). Brief Compendium of Medicinal Herbs (*Yakuso Rhakuhu*: 薬草略譜). Tokyo: National Diet Library Digital Collections, 54. https://dl.ndl.go.jp/pid/2535717

Kyushu Medicinal Plant Research Society (1929). Medicinal Plants for Home Knowledge (家庭須知薬用植物). Tokyo: National Diet Library Digital Collections, 152. https://dl.ndl.go.jp/pid/1057198

Li, S.Z. (2004). Rpt. Compendium of Materia Medica (本草綱目) 2nd. Ed. Beijing: People's Medicinal Publishing House.

Makino, T. (1896). *Rumex madaio*. *The Botanical Magazine***,** 107–108. https://www.biodiversitylibrary.org/item/129783

Makino, T. (1940). Illustrated Flora of Nippon, with the Cultivated and Naturalized Plants by Tomitaro Makino (牧野日本植物図鑑). Tokyo: Hokuryukan, 630–632. http://www.hokuryukan-ns.co.jp/makino/

Manase, G. (1698). Augmented Edition of the Lingbao Medicinal Properties, Efficiency, and Toxicity (*Zoho Reiho Yakusho Nodoku*: 増補霊寳薬性能毒).Tokyo: Waseda University Library's collection of Japanese and Chinese classics, 8–12. https://archive.wul.waseda.ac.jp/kosho/bunko31/bunko31_e1592/bunko31_e1592_0003/bunko31_e1592_0003.pdf

Maruyama, F. (1923). Concise Lectures for Drug Merchants (簡明薬種商講義). Tokyo: National Diet Library Digital Collections, 171–172. https://dl.ndl.go.jp/pid/931707

Masatsugu, S. (1712). Materia Medica Harmony and Interpretation (*Honzo Wage*: 本草和解). Tokyo: National Diet Library Digital Collections, 8–12. https://dl.ndl.go.jp/pid/2557701

Matsuda, H. (1920). Cultivation of Medicinal Plants for New Drugs (新薬植物栽培). Tokyo: National Diet Library Digital Collections, 74–75. https://dl.ndl.go.jp/pid/931664

Matsuoka, G. (1726). Essential Knowledge for Using Medicines (*Yoyaku Suchi*: 用薬須知). Tokyo: National Diet Library Digital Collections, 22. https://dl.ndl.go.jp/pid/2536607

Miyashita, S. (2018). Manuscripts on imported drugs and kitchenwares the later Edo period. *Bull. Inst. Orient. Occid. Stud. Kansai Univ.* 28, 31–62. https://kansai-u.repo.nii.ac.jp/records/1685

Miyazaki, Y. (1697). Complete Book of Agriculture (*Nogyo Zensho*: 農業全書). Tokyo: National Diet Library Digital Collections, 19–20. https://dl.ndl.go.jp/pid/2558577

Mizutani, T. (Unknown year). Recorded Hearings on the Compendium of Materia Medica (*Honzo Komoku Kibun*: 本草綱目紀聞). Tokyo: National Diet Library Digital Collections, 5–9. https://dl.ndl.go.jp/pid/2580162

Murakoshi, M. (1952). llustrated Guide to Medicinal Plants (薬用植物図説). Tokyo: National Diet Library Digital Collections, 27–28. https://dl.ndl.go.jp/pid/2423405

Nagao, M. (1925). Modern Medicines in Clinical Practice (臨床近世薬物) Fifth Edition. Tokyo: National Diet Library Digital Collections, 108–110. https://dl.ndl.go.jp/pid/931530

Nagao, O. (1924). The Japanese Pharmacopoeia Fourth Edition for Physicians with Prescriptions (処方添註医家用第四改正日本薬局方). Tokyo: National Diet Library Digital Collections, 313. https://dl.ndl.go.jp/pid/931708

Naito, H. (1840). Investigation of Crude Drugs in Classical Prescriptions (*Koho Yakuhin Ko*: 古方薬品考). Tokyo: National Diet Library Digital Collections, 122–124. https://dl.ndl.go.jp/pid/2536679

Nakajima, T. (1911). Essentials of Pharmacognosy (生薬学要訣). Tokyo: National Diet Library Digital Collections, 38–39. https://dl.ndl.go.jp/pid/837468

Nakayama, T. (1929). Supplementary Notes on Kampo Medicines (漢方医学余談). Tokyo: National Diet Library Digital Collections, 82. https://dl.ndl.go.jp/pid/1049064

Nishimura, T. (1911). Pocket Pharmacognosy (ポケット生薬学). Tokyo: National Diet Library Digital Collections, 33. https://dl.ndl.go.jp/pid/837650

Oguchi, Y. (1754). Clarifying Confusions about Crude Drugs (*Yakuhin Benwaku*: 薬品弁惑). Tokyo: National Diet Library Digital Collections, 16. https://dl.ndl.go.jp/pid/2536583

Okamoto, I. (1698). Complete Compendium of Materia Medica of Wide Benefit (*Koeki Honzo Taisei*: 広益本草大成). Tokyo: National Diet Library Digital Collections, 20–23. https://dl.ndl.go.jp/pid/2557115

Okamoto, I. (1699). Explanation and Commentary on Records of Medicinal Properties (*Yakushoki Benkai*: 薬性記弁解). Kyoto: Kyoto University Rare Materials Digital Archive, 62–64. https://rmda.kulib.kyoto-u.ac.jp/item/rb00005434

Okunishi, T. (1710). Record of Newly Formulated Preparations of Medicinal Varieties (*Yakushu Shin Seizaiki*: 薬種新製剤記). Tokyo: National Diet Library Digital Collections, 3. https://dl.ndl.go.jp/pid/2557572

Onda, S. (1918). Annotated Complete Collection for Pharmacist Examination Questions and Answers Seventh Edition (説明附薬剤師試験問題答案全集) Tokyo: National Diet Library Digital Collections, 252–254. https://dl.ndl.go.jp/pid/931574

Ono, R. (1805). Elucidation of Compendium of Materia Medica (*Honzo Komoku Keimo*: 本草綱目啓蒙). Tokyo: National Diet Library Digital Collections, 4–6. https://dl.ndl.go.jp/pid/2555480

Ono, R. (Unknown year). Recorded Hearings on Materia Medica (*Honzo Kibun*: 本草記聞). Tokyo: National Diet Library Digital Collections, 4–6. https://dl.ndl.go.jp/pid/2556936

Ota, Y. (1878). Outline of Materia Medica (薬物学大意). Tokyo: National Diet Library Digital Collections, 12–14. https://dl.ndl.go.jp/pid/837756

Pharmaceutical and Medical Device Regulatory Science Society of Japan (1961). The Japanese Pharmacopoeia Seventh Edition. Tokyo: Hirokawa Publishing Company, 323–324.

Romaniello, M.P. (2016). True rhubarb? Trading Eurasian botanical and medical knowledge in the eighteenth century. *J. Glob. Hist.* 11, 3–23. doi:10.1017/S1740022815000327

Ryosaku, E. (1925). Detailed Explanations of New Medicines (新薬詳解). Tokyo: National Diet Library Digital Collections, 430. https://dl.ndl.go.jp/pid/931719

Saita, K. (1897). General Flora of Great Japan (大日本普通植物誌). Tokyo: National Diet Library Digital Collections, 147. https://dl.ndl.go.jp/pid/832549

Saito, N. (Unknown year). Mirror of Materia Medica (*Honzo Kagami*: 本草鏡). Tokyo: National Diet Library Digital Collections, 4–5. https://dl.ndl.go.jp/pid/2557986

Sano, T. (1889). Practical Methods for Medicine Identification (実用薬品鑑定法). Tokyo: National Diet Library Digital Collections, 82. https://dl.ndl.go.jp/pid/837438

Shibata, M. (1810). Study of Crude Drugs for Daily Use (*Nichiyo Yakuhin Ko*: 日用薬品考). Tokyo: Waseda University Library's collection of Japanese and Chinese classics, 51–52. https://dl.ndl.go.jp/info:ndljp/pid/2536868

Shimazaki, K. (1909). Concise Pharmacognosy (簡明生薬学). Tokyo: National Diet Library Digital Collections, 60–61. https://dl.ndl.go.jp/pid/837391

Shimizu, N. (1859). Study of Japanese Rhubarb (*Nihon Daioko*: 日本大黄考). Tokyo: National Diet Library Digital Collections. https://dl.ndl.go.jp/pid/2536361

Shimizu, T. (1941). Studies on Japanese Medicines (国医薬物学研究). Tokyo: Hirokawa Publishing Company, 142–144.

Shimotsu, M. (1680). Illustrated Materia Medica (*Zukai Honzo*: 図解本草). Tokyo: National Diet Library Digital Collections, 5–8. https://dl.ndl.go.jp/pid/2556850

Shimoyama, T. (1943). Pharmacognosy (生薬学) 27th Edition. Tokyo: Syoubunsya inc., 111–114.

Shimoyama, Z. (1904). Pharmacognosy (生薬学) Sixth Edition. Tokyo: National Diet Library Digital Collections, 50–53. https://dl.ndl.go.jp/pid/837458

So, H. (1798). Compilation and Commentary on the Compendium of Materia Medica (*Honzo Komoku Sanso*: 本草綱目纂疏). Tokyo: National Diet Library Digital Collections, 6–7. https://dl.ndl.go.jp/pid/2580137

Sugiyama, N., and Suda, K. (1891). Commentary on the Japanese Pharmacopoeia (日本薬局方註釈) Tokyo: National Diet Library Digital Collections, 272–273. https://dl.ndl.go.jp/pid/837903/1/273

Takada, G. (1683). Materia Medica of Decoctions and Precious Fragments (*Toeki Hengyoku Honzo*: 湯液片玉本草). Tokyo: National Diet Library Digital Collections, 89. https://dl.ndl.go.jp/pid/2536852

Tashiro, K. (1999). Research on the Investigation of Korean Medicinal Materials in the Edo Period (江戸時代朝鮮薬材調査の研究). Tokyo: Keio University Press.

Terashima, R. (1824). llustrated Sino-Japanese Encyclopedia of the Three Realms (*Wakan Sansai Zue*: 和漢三才図会). Tokyo: National Diet Library Digital Collections, 4–5. https://dl.ndl.go.jp/pid/2569770

Udagawa, S. (1830). Newly Revised and Augmented Dutch Mirror of Medicine (*Shintei Zoho Oranda Yakkyo*: 新訂増補和蘭薬鏡). Tokyo: National Diet Library Digital Collections, 3–13. https://dl.ndl.go.jp/pid/2555324

Ujita, I. (1795). Explanations of Crude Drugs in Classical Prescriptions (*Koho Yakusetsu*: 古方薬説). Tokyo: National Diet Library Digital Collections, 19–20. https://dl.ndl.go.jp/pid/2556026

Unknown Author (1826). Secret Formulas of Materia Medica (*Honzo Hiketsu*: 本草秘訣). Tokyo: National Diet Library Digital Collections, 2–3. https://dl.ndl.go.jp/pid/2558004

Wigand, A. (1888). Pharmacognosy (生薬学), translated by Oi, G. Tokyo: National Diet Library Digital Collections, 43–46. https://dl.ndl.go.jp/pid/837455

Yakugyo Jiho Co. (1932). The Japanese Pharmacopoeia Fifth Edition. Tokyo: National Diet Library Digital Collections, 185. https://dl.ndl.go.jp/pid/1025529

Yoshimatsu, I. (1947). Latest Classified Compilation of Pharmaceutical Products (最新医薬品類聚). Tokyo: National Diet Library Digital Collections, 141–145. https://dl.ndl.go.jp/pid/1045801
